# Shedding light on imagined scents: an fNIRS feasibility study of olfactory mental imagery and semantic cueing

**DOI:** 10.3389/fnins.2026.1825171

**Published:** 2026-08-10

**Authors:** Andrew Levy, Eleanor Boot, Muriel Jacquot, Giuliano Gaeta, Emilia Parkkinen, Tyler Mari, Ilias Tachtsidis

**Affiliations:** 1 Metabolight Ltd, London, United Kingdom; 2 Department of Imaging Neuroscience, University College London, London, United Kingdom; 3 Givaudan UK Ltd, Ashford, United Kingdom; 4 Department of Medical Physics & Biomedical Engineering, University College London, London, United Kingdom

**Keywords:** color-odor association, correlation-based signal improvement, functional near-infrared spectroscopy, mental imagery, multisensory imagery, olfaction, olfactory imagery, orbitofrontal cortex

## Abstract

Mental imagery is the ability to evoke sensory experiences without a corresponding stimulus, with established roles in memory, cognition, and behavior across multiple sensory modalities. Whilst visual and auditory imagery have been extensively characterized using neuroimaging, olfactory imagery remains comparatively understudied despite representing a particularly compelling target for investigation. Unlike other sensory systems, olfactory information projects directly to limbic structures including the amygdala and hippocampus without thalamic relay, providing a neurobiological basis for the characteristically vivid and emotionally evocative quality of odor-related memories and imagery. Understanding the cortical correlates of olfactory imagery therefore holds both theoretical and applied relevance, with implications for consumer neuroscience, food science, and the study of memory and multisensory cognition. A key methodological challenge is that primary olfactory structures reside in deep subcortical regions inaccessible to fNIRS, yet higher-order olfactory regions including the orbitofrontal cortex fall within its measurement depth. We therefore conducted a feasibility study evaluating whether functional near-infrared spectroscopy (fNIRS) can detect cortical signatures of olfactory mental imagery in these accessible regions. Thirty-five participants were recruited, of whom 30 were included in the final analysis. Participants generated visual or olfactory mental images in response to semantic cues (odor-related words) or perceptual cues (abstract color patterns) in a 2x2 factorial design. Cortical haemodynamic responses were measured using a 42-channel fNIRS array covering frontal, temporal, and parietal regions, with data analyzed using oxyhaemoglobin (HbO), deoxyhaemoglobin (HbR), and correlation-based signal improvement (CBSI), and group-level inference performed using surface-based statistical parametric mapping. Visual imagery produced robust bilateral frontoparietal activation across all chromophores, validating the experimental framework. Olfactory imagery produced modest bilateral orbitofrontal activation, detected exclusively in HbO measurements, providing tentative preliminary evidence that fNIRS can detect higher-order olfactory imagery-related cortical activity. Post-hoc chromophore analysis revealed striking discordance across conditions, with only visual imagery to words producing concordant HbO and HbR responses surviving CBSI correction, and deactivations detected exclusively in HbO. These findings offer qualified support for the feasibility of fNIRS in this domain whilst raising important questions about chromophore coupling dynamics during endogenously driven cognitive processes and the suitability of CBSI assumptions beyond conventional stimulus-driven paradigms.

## 1 Introduction

Mental imagery is the capacity to evoke sensory experiences in the absence of corresponding stimuli, representing a fundamental aspect of human cognition with established roles in working memory (Pearson et al., 2015), memory consolidation (Marre et al., 2021; Lenormand et al., 2024), spatial reasoning (Borst, 2013), and social cognition (Zagacki et al., 1992; Zhang et al., 2025). The capacity for mental imagery extends across multiple sensory modalities, with visual and auditory experiences representing particularly vivid forms of imagination alongside olfactory, gustatory, tactile, and motor imagery (McNorgan, 2012; Van Caenegem et al., 2024). Whilst visual (Pearson, 2019) and auditory (Zvyagintsev et al., 2013) imagery have been extensively characterized using neuroimaging methods, olfactory imagery remains comparatively understudied (Boot et al., 2024; Spence, 2024). This is despite accumulating evidence suggesting distinct neural correlates detectable with non-invasive neuroimaging (Han et al., 2022). The present study evaluates whether functional near-infrared spectroscopy (fNIRS) can detect cortical signatures of olfactory imagery despite the technique's inability to access primary olfactory structures in piriform cortex and medial temporal lobe, and in doing so examines the feasibility of investigating olfactory cognition in naturalistic contexts where traditional neuroimaging methods remain impractical.

Applying fNIRS to olfactory imagery faces a fundamental methodological challenge because the primary olfactory cortices lie in subcortical and medial temporal structures. The piriform cortex, amygdala, and anterior hippocampus lie more than 3 to 4 cm below the scalp, far deeper than the roughly 1.5 cm that fNIRS can typically reach. An additional factor is the wide variability in olfactory imagery ability across individuals. Unlike visual imagery, where only a few people report being unable to form mental images, many people struggle or fail to evoke smells in their minds (Morrot et al., 2013; Bensafi and Rouby, 2007). This variability is influenced by semantic knowledge (Palmiero et al., 2014), perceptual experience (Arshamian and Larsson, 2014), and olfactory expertise (Royet et al., 2013), suggesting that olfactory imagery depends on learning and experience.

Despite these apparent limitations, fNIRS offers compelling advantages for imagery research that may offset its restricted spatial coverage. The technique's exceptional tolerance to motion artifacts enables data collection in naturalistic settings (Pinti et al., 2018; Gaeta et al., 2024), a consideration of particular relevance for mental imagery paradigms where ecological validity and participant comfort may influence imagery vividness (Magalhães et al., 2025). Moreover, fNIRS systems permit extended recording periods without the acoustic noise, spatial confinement, and postural restrictions inherent to fMRI, potentially yielding more representative measurements of imagery-related brain activity. Crucially, whilst primary olfactory structures remain inaccessible to fNIRS, higher-order cortical regions implicated in olfactory imagery, including the orbitofrontal cortex, inferior frontal gyrus, and inferior parietal cortex, fall within the measurement depth of fNIRS and may exhibit differential activation during olfactory compared to visual imagery.

Olfactory imagery is theoretically important because it provides a way to examine how odor representations can be internally reactivated in the absence of physical stimulation. This is particularly relevant because olfaction differs from vision and audition in both neuroanatomy and phenomenology. Unlike other sensory systems, olfactory information reaches limbic and medial temporal structures, including the amygdala and hippocampus, with limited thalamic mediation, providing a neurobiological basis for the close links between odor, emotion, memory, and autobiographical recall (Kay and Sherman, 2007; Shepherd, 2005). Olfactory imagery, therefore, offers an informative case for studying how sensory, affective, and mnemonic representations are reconstructed endogenously. Olfactory imagery can also be evoked through indirect cues rather than through odors themselves. Odor-related words can trigger olfactory mental images by engaging semantic knowledge and prior perceptual experience (Köster et al., 2014; Arshamian et al., 2008), whereas abstract color arrangements may act as non-verbal crossmodal cues based on learned or culturally shared odor-color correspondences (Spence, 2020; Hossu et al., 2024). In the present study, color patches were therefore used as perceptual supports for olfactory imagery rather than as arbitrary visual stimuli. This cueing logic is relevant beyond the laboratory, because everyday olfactory expectations are often elicited by language, packaging, color, and visual context before any odor is physically present. Functional neuroimaging studies using fMRI and PET indicate that cued olfactory imagery can engage regions involved in odor processing, including orbitofrontal cortex, piriform cortex, insula, and hippocampus (Torske et al., 2022), providing a rationale for testing whether higher-order cortical components of this process can be detected with fNIRS.

The present study is a feasibility investigation with the primary aim of establishing whether fNIRS can detect cortical signatures of olfactory imagery despite the technique's inability to access primary olfactory structures. We employed a factorial design that contrasted olfactory and visual imagery, with each modality evoked by two stimulus types, namely odor-related words and abstract color patches. We focused our analyses on accessible cortical regions implicated in higher-order olfactory processing, including orbitofrontal cortex and inferior frontal gyrus, where olfactory imagery effects might reasonably be detected. By systematically comparing olfactory and visual imagery under matched experimental conditions, we aimed to establish whether fNIRS provides sufficient sensitivity to detect modality-specific cortical contributions to mental imagery. Successful demonstration of such effects would validate fNIRS as a viable tool for investigating olfactory cognition in naturalistic contexts where traditional neuroimaging methods remain impractical, opening new avenues for olfactory neuroimaging research in individual differences, consumer behavior, and multisensory experience.

## 2 Methods

### 2.1 Participants

Participants were recruited by the Givaudan Sensory Science Department via an external participant pool. The study was approved by Givaudan's Internal Review Board, ethics no. 2019-002 and all participants provided written informed consent prior to taking part. Participants received compensation for their time.

Thirty-five participants were recruited in total (28 females and 7 males; aged 18–50, mean 38.52 ± 7.83 years), a sample considered appropriate for a feasibility study of this kind and exceeding those typically employed in comparable fNIRS investigations. No formal power analysis was conducted, as no prior effect size estimates exist for fNIRS-based olfactory imagery research (Yücel et al., 2021). Inclusion criteria required participants to be aged 50 or under, report no olfactory dysfunction, and be healthy with no recent history of cold, flu, or respiratory virus, including COVID-19. Prior to the study, participants completed the Vividness of Visual Imagery Questionnaire [VVIQ; Marks (1973)] and the Vividness of Olfactory Imagery Questionnaire [VOIQ; Gilbert et al. (1996)] as pre-screening measures. For the VVIQ, scores below 23 indicate aphantasia (absence of visual imagery), while scores above 75 indicate hyperphantasia (exceptionally vivid imagery). No participants met the criteria for aphantasia, and two participants scored within the hyperphantasia range. There are currently no established clinical reference ranges for VOIQ scores, reflecting the relative novelty of systematic olfactory imagery assessment. No participants met exclusion criteria on either measure. Five participants were excluded from analysis due to data acquisition errors arising from device malfunction during recording, leaving a final analyzed sample of 30 participants.

### 2.2 Experiment

#### 2.2.1 Task design

**Figure 1:**
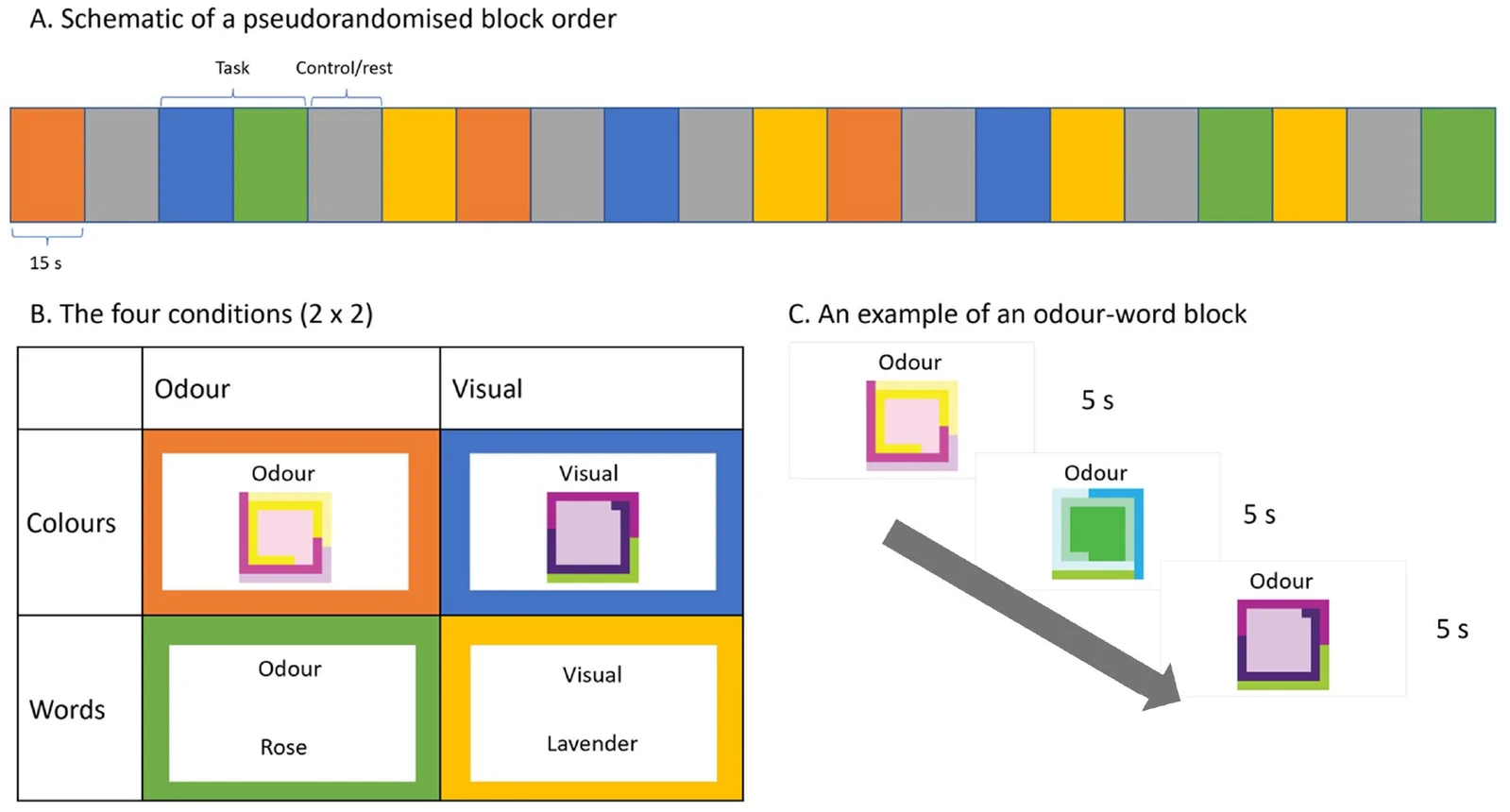
Experimental task design: **(A)** Schematic of the pseudorandomised block structure showing the alternation between task blocks (colored) and control/rest periods (gray) across a 15-second cycle. Each color represents a different experimental condition. **(B)** The 2 × 2 factorial design with Imagery Modality (olfactory versus visual) and Representation Type (color patterns versus words) as factors, resulting in four conditions: olfactory imagery with color patterns (OI-C), visual imagery with color patterns (VI-C), olfactory imagery with words (OI-W), and visual imagery with words (VI-W). **(C)** Example of stimulus presentation within an odor-word block, showing three sequential 5-second trials in which participants generate mental images of the cued modality (odor) based on the presented stimuli (chromatic patterns representing rose, peppermint, and lavender).

The task employed a within-subjects repeated-measures 2 × 2 factorial design, with all participants completing all four experimental conditions. Imagery Modality (visual versus olfactory) and Cue Representation Type (verbal versus color patterns) served as within-subjects factors, resulting in four experimental conditions: visual imagery with color patterns (VI-C), visual imagery with words (VI-W), olfactory imagery with color patterns (OI-C), and olfactory imagery with words (OI-W; see Figure 1B). The task was built and run using the Gorilla Experiment Builder (Anwyl-Irvine et al., 2020), a cloud-based platform. On each trial, participants viewed a screen displaying an instruction of either ‘Visual' or ‘Olfactory' at the top and either a word or color-based imagery cue at the bottom, with a fixation cross persisting throughout. Participants were instructed to form a mental image related to the imagery cue in the specified modality. Each screen was displayed for 5,000 ms with a 500 ms interval, such that three stimuli were presented within each 16-second block (Figure 1A). All presentations within a block were from the same imagery modality and stimulus type. Task blocks were presented in a pseudorandomised order to prevent two consecutive identical block types (Figure 1C), with jittered rest periods of 14 to 18 seconds interleaved between blocks. Rest periods occurred after one to two task blocks, during which participants saw a fixation cross and were instructed to breathe normally. The experiment comprised 36 stimulus presentations across 12 blocks per condition, totalling 48 task blocks. The task was divided into three experimental runs with two break periods. Three pseudorandomised task orders were established, and participants were randomly assigned to one. The experiment lasted approximately 20 minutes, including breaks.

#### 2.2.2 Color pattern generation

Color-pattern stimuli were generated using a proprietary computational procedure used to transform odor-color association profiles into abstract chromatic patterns. Because the generation algorithm is proprietary, the full implementation cannot be disclosed or redistributed. However, the present experiment relies on the final visual stimuli presented to participants rather than on access to the generation algorithm. To ensure reproducibility of the experimental materials, the final color-pattern stimuli are fully specified in Supplementary Table S1.

**Figure 2:**
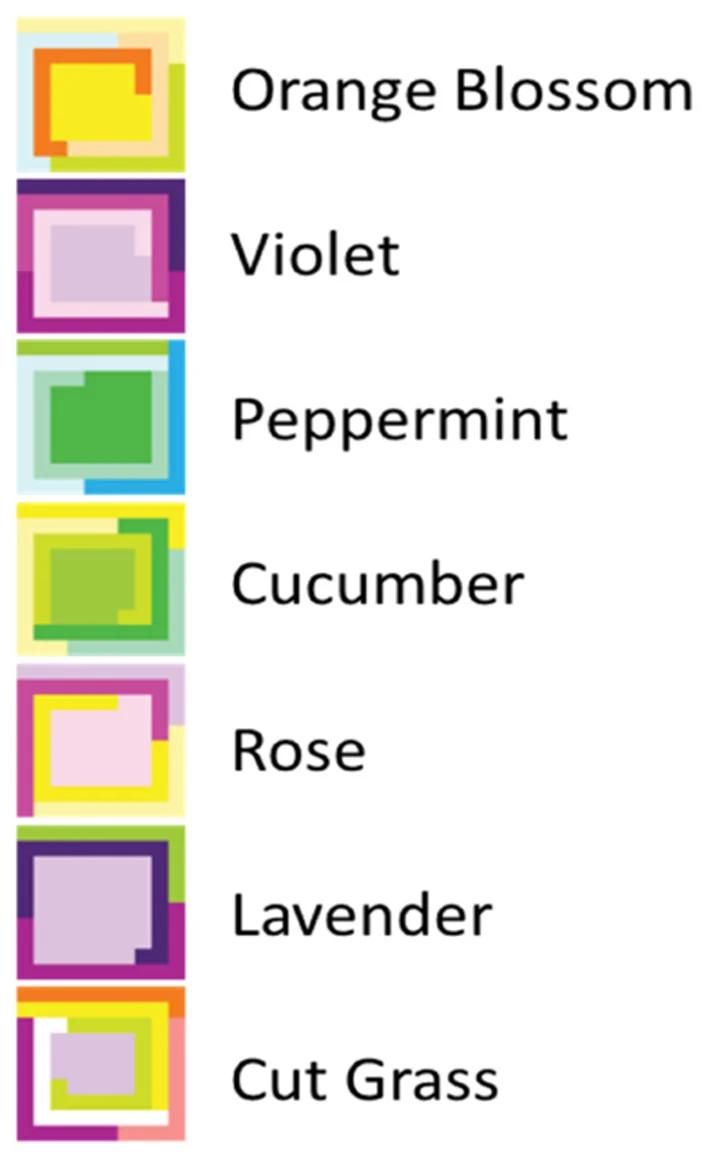
Examples of chromatic patterns generated for seven odorants: Chromatic patterns generated for the target odorants. Each pattern comprises multiple color patches arranged in an abstract spatial configuration. Patch size reflects the relative weight of each color association for the corresponding odorant. Exact HEX and 8-bit sRGB values and relative patch areas for the final displayed stimuli are provided in Supplementary Table S1.

In brief, each target odorant was associated with a multi-color profile derived from empirical odor-color association data. The final stimuli consisted of abstract patterns composed of multiple color patches, with patch size reflecting the relative weight of each color association for the corresponding odorant (Figure 2). The same color-pattern stimuli were used in both color-cued imagery conditions.

Colors were specified using HEX codes and 8-bit sRGB values, with each RGB channel ranging from 0 to 255. For each color-pattern stimulus, Supplementary Table S1 reports a preview of the pattern, the exact HEX and RGB values of each color patch, and the relative area occupied by each color within the final displayed stimulus. Relative areas sum to 100% for each target odorant. These specifications allow the final experimental stimuli to be reconstructed independently of the proprietary generation algorithm.

#### 2.2.3 Vividness questionnaire

Individual differences in imagery ability were assessed using the Vividness of Visual Imagery Questionnaire [VVIQ; Marks (1973)] and Vividness of Olfactory Imagery Questionnaire [VOIQ; Gilbert et al. (1996)]. Both instruments comprise 16 items across four scenarios, with participants rating imagery vividness on 5-point scales from “no image at all” to “perfectly realistic, as vivid as real seeing/smelling”. The VVIQ assesses visual imagery of common scenes (e.g., “the sun sinking below the horizon”), whilst the VOIQ employs an identical structure for olfactory imagery (e.g., “the smell of frying bacon”). Both questionnaires demonstrate high internal consistency and test-retest reliability (Campos and Pérez-Fabello, 2009; Kollndorfer et al., 2015; Fantin et al., 2022), with scores correlating with neural activation in modality-specific sensory cortices during imagery tasks (Cui et al., 2007; Djordjevic et al., 2005). These measures were included as covariates in subsequent analyses to account for individual variance in imagery capacity.

### 2.3 Data acquisition

#### 2.3.1 fNIRS

**Figure 3:**
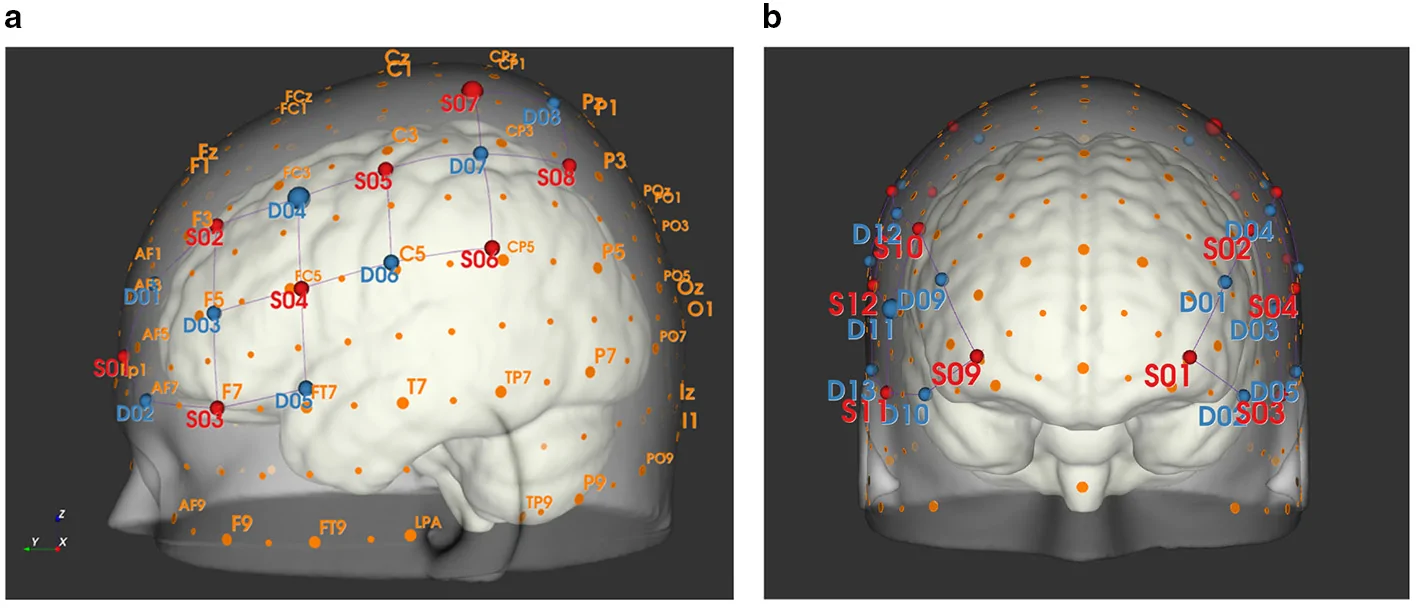
fNIRS optode montage: Sixteen sources (red) and 16 detectors (blue) were positioned across frontal, central, and parietal regions to create 42 channels. **(a)** Left hemisphere view: 8 sources and 8 detectors forming 21 channels, registered to 10–20 system landmarks (orange labels). **(b)** Anterior view: optode positions in relation to 10-20 system (orange labels).

Scanning was conducted using the NirSport 2 (NIRx, USA). Continuous-wave fNIRS recordings were captured using 760 nm and 850 nm wavelengths at a sampling frequency of 7.63 Hz, and acquired using NIRx Aurora software (version 2021.9). Scanning was performed across a 42-channel custom array. The array was designed using NIRx NirSite 2021.4. A previous review of the literature identified key regions of interest (ROIs) for monitoring olfactory imagery using fNIRS (Boot et al., 2024). 16 sources and 16 detectors were arranged at 10–20 locations to provide coverage of identified ROIs (Figure 3). Full optode MNI coordinates are provided in Supplementary Table S2.

#### 2.3.2 Systemic monitoring

Physiological data were acquired using an in-house developed non-invasive wearable patch positioned on the thorax, providing continuous recordings of electrocardiographic (ECG) activity and galvanic skin response (GSR) at a sampling rate of 400 Hz.

### 2.4 fNIRS preprocessing

**Figure 4:**
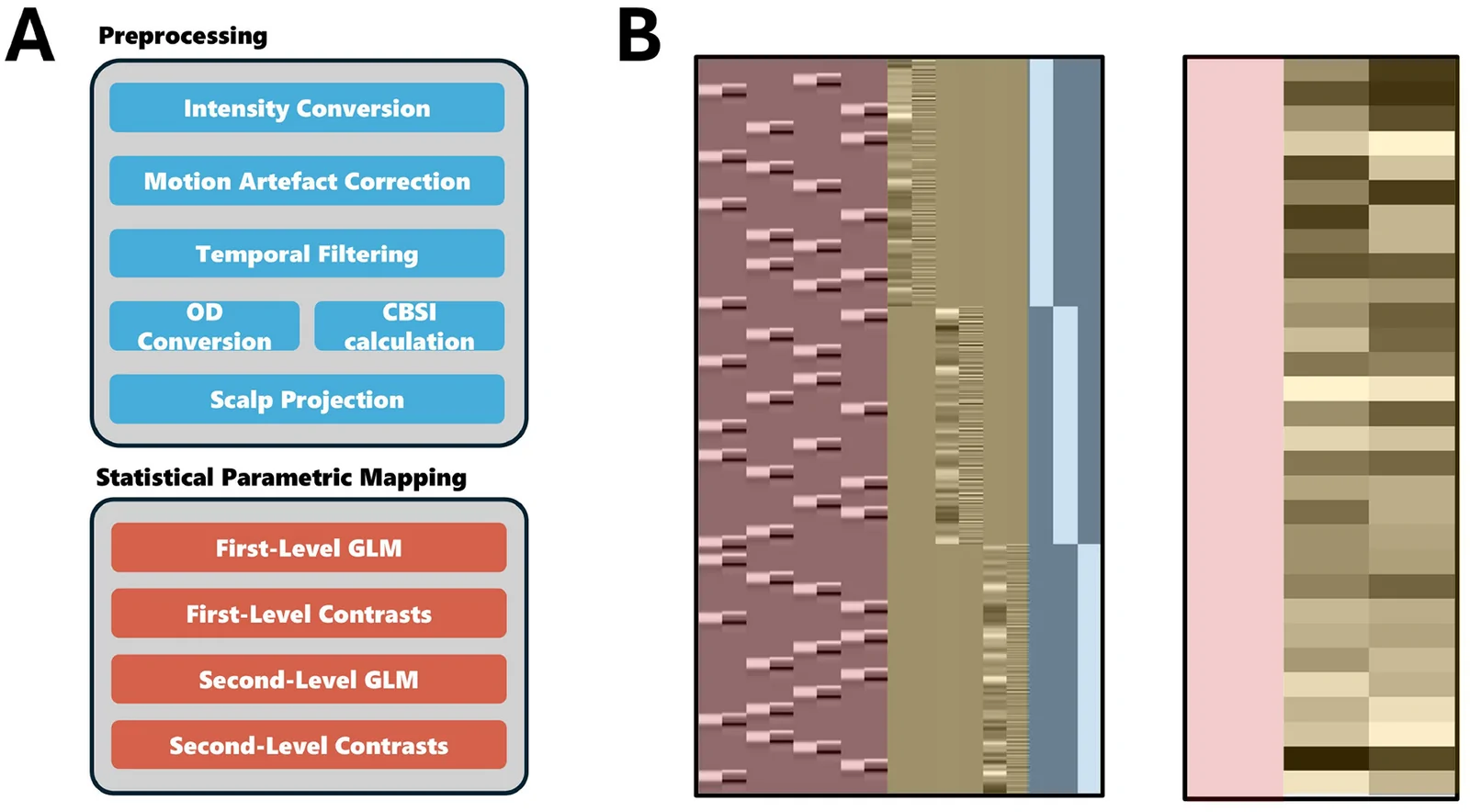
Statistical analysis pipeline and design matrices: **(A)** The preprocessing pipeline applies intensity conversion, motion artifact correction, temporal filtering, optical density conversion with CBSI calculation, and scalp projection to prepare the fNIRS data. Statistical parametric mapping then proceeds through first-level GLM analysis to estimate task-related effects for each participant, first-level contrasts to compare experimental conditions, second-level GLM to assess group-level effects, and second-level contrasts to test population-level hypotheses. **(B)** Design matrices showing the structure of the statistical models. Left panel: First-level GLM design matrix for a representative participant. Experimental regressors (red/pink) model the four conditions (visual imagery with words, visual imagery with color patches, olfactory imagery with words, and olfactory imagery with color patches), each convolved with the canonical haemodynamic response function and its temporal derivative. These regressors are concatenated across the three experimental runs to improve parameter estimation. Nuisance regressors (yellow/brown) comprise heart rate and galvanic skin response convolved with the cardiac response function, modeled separately for each run and orthogonalised against experimental effects to remove systemic physiological noise whilst preserving task-related variance. Constant terms (blue) model session-specific baseline levels. Right panel: Second-level GLM design matrix for group inference. The model comprises a group mean (intercept) and covariates for individual imagery ability measured by the Vividness of Visual Imagery Questionnaire (VVIQ) and Vividness of Olfactory Imagery Questionnaire (VOIQ, reverse-scored so that higher scores reflect greater vividness rather than greater dysfunction, as originally designed), allowing estimation of population-level effects whilst accounting for inter-individual differences in subjective imagery capacity.

fNIRS data were preprocessed and analyzed using SPM 25.01.02 (Friston et al., 1994), FieldTrip (2024 release) (Oostenveld et al., 2011), Homer3 v1.87.0 (Huppert et al., 2009), and custom MATLAB scripts, all running under MATLAB R2023b. Custom scripts were developed specifically to project haemodynamic data onto a canonical scalp surface mesh, facilitating mass-univariate inference within SPM and enabling familywise error correction via random field theory. A visual overview of the complete preprocessing and analysis pipeline is provided in Figure 4A, and each step is detailed in turn below.

**Quality Inspection:** Signal quality was assessed via visual inspection of raw channel data by an experienced fNIRS user. Channels were deemed acceptable if they exhibited a clear, discernible cardiac peak at approximately 1 Hz in the power spectral density, indicative of adequate scalp coupling, alongside the absence of white noise characteristics associated with flat or poorly coupled channels and the absence of excessive motion-related artifacts. Participants with fewer than 80% acceptable channels were excluded (*n* = 0).

**Spatial Registration:** Optode and channel positions were transformed to MNI space using two-stage rigid registration. Anatomical fiducials (nasion, inion, preauricular points) from the digitized montage were aligned to standard SPM coordinates, followed by iterative closest point (ICP) registration between the headshape point cloud and the canonical scalp surface.

**Intensity Conversion:** Raw light intensity data were converted to optical density as *OD* = −log(*I*/*I*_0_), where *I* represents the raw intensity and *I*_0_ the temporal mean.

**Motion Artifact Correction:** Motion artifacts were corrected using a wavelet-based approach implemented in the Homer3 toolbox (Molavi and Dumont, 2012). The method employed discrete wavelet decomposition with a Daubechies-2 wavelet basis to identify and suppress motion-corrupted segments based on interquartile range thresholding (IQR = 2.2). No accelerometer data were acquired during the study; any residual head movement not captured by the wavelet correction therefore, represents a potential minor source of noise, though this is considered minimal given the low-motion seated nature of the paradigm.

**Low-pass Filtering:** Optical density data were filtered using a fifth-order Butterworth low-pass filter with a cut-off frequency of 1 Hz, applied bidirectionally to prevent temporal distortions. Whilst this cutoff is liberal relative to field convention (Pinti et al., 2019), it was chosen deliberately to preserve sufficient spectral content for stable AR coefficient estimation during prewhitening, as low-pass filtering applied prior to AR-based prewhitening can itself introduce serial correlations into the residuals, constituting new violations of the statistical model assumptions (Huppert, 2016).

**Conversion:** Optical density data were converted to oxyhaemoglobin (HbO), deoxyhaemoglobin (HbR) using the modified Beer-Lambert law with wavelength-specific extinction coefficients and differential pathlength factors (DPF = 6).

**CBSI:** Correlation-based signal improvement (CBSI) was applied to reduce systemic physiological noise and motion artifacts (Cui et al., 2010). The CBSI signal was calculated for each channel as *CBSI* = 0.5(*HbO* − α·*HbR*), where α is the ratio of standard deviations (σ_*HbO*_/σ_*HbR*_), exploiting the negative correlation between hemoglobin species during systemic interference whilst preserving the positive correlation during neuronal activation.

**Downsampling:** Data were downsampled to 1 Hz using the resample method, which applies anti-aliasing filtering prior to decimation to prevent spectral aliasing.

#### 2.4.1 Scalp projection

**Figure 5:**
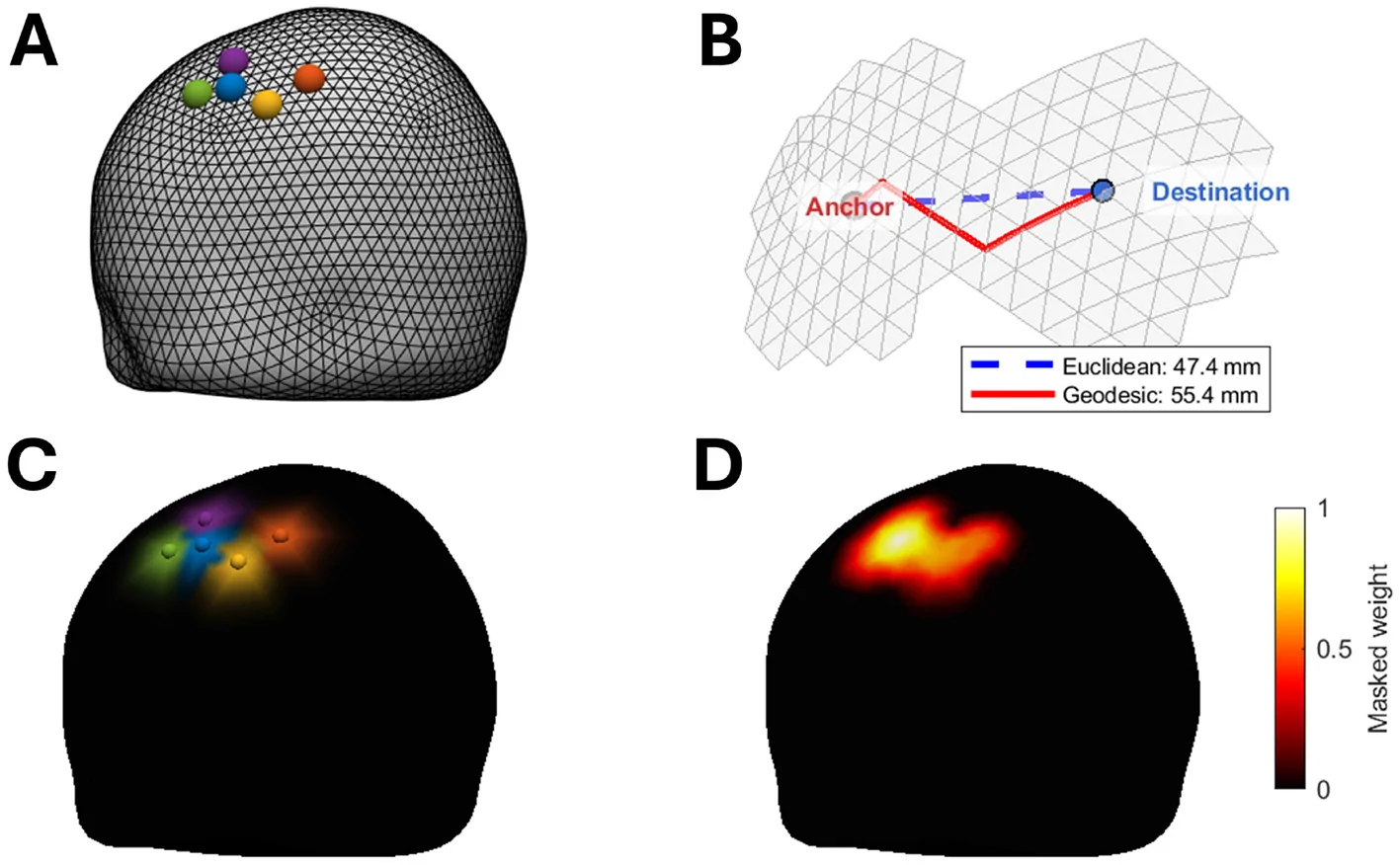
Geodesic Gaussian interpolation for scalp projection: **(A)** Canonical scalp mesh (2,562 vertices) with fNIRS channel anchor positions shown as colored spheres. Each channel is mapped to its nearest mesh vertex, providing a standardized coordinate system for cross-subject analysis. **(B)** Comparison of Euclidean (blue dashed line) versus geodesic (red solid line) distance computation between an anchor and destination vertex. The geodesic path respects the curved scalp geometry, yielding distances that accurately reflect true surface separation—particularly important over highly curved regions where Euclidean distances underestimate surface travel. **(C)** Fixed-width Gaussian kernel weights (FWHM = 30 mm) radiating from each anchor. Each anchor's influence is represented by a distinct color, with weights decaying exponentially as a function of geodesic distance. The fixed kernel width ensures approximately constant spatial smoothness across the surface, satisfying stationarity assumptions required for valid random field theory-based inference. **(D)** Application of the cosine-tapered boundary mask to prevent extrapolation artifacts. The mask transitions smoothly from full weight (yellow/white) to zero (black) at the periphery of the measurement domain, ensuring differentiability whilst restricting inference to regions with direct fNIRS coverage.

Channel-level haemodynamic data were projected onto a canonical scalp surface mesh prior to statistical analysis using a custom geodesic-based interpolation procedure implemented within SPM (Friston et al., 2007), enabling mass-univariate inference with random field theory-based familywise error correction. This approach is conventional within the MEG/EEG literature (Mattout et al., 2007) and extends its application to fNIRS, offering an advantage over previously implemented SPM-based fNIRS analyses by preserving geodesic rather than Euclidean distances across the scalp surface, ensuring geometric fidelity in curved regions such as the temporal lobes. By mapping individual participant data onto a canonical scalp mesh, the procedure enables vertex-wise group statistics at corresponding scalp positions whilst accounting for individual variation in head geometry and optode placement. A cosine-tapered mask was applied at ROI boundaries to prevent extrapolation artifacts in regions distant from fNIRS channels. The full technical specification of the procedure, including the interpolation equations and relevant code, is provided in Supplementary material. The key stages of the procedure are illustrated in Figure 5.

This combined framework of geodesic distance metrics, fixed-width Gaussian kernels, cosine-tapered boundary masking, and canonical mesh provides a principled and statistically valid approach for group-level inference. The method is directly compatible with SPM's established inference machinery, including parametric statistical inference with random field theory-based correction for multiple comparisons at the vertex level. Moreover, by avoiding unconstrained extrapolation beyond the convex hull of channel positions (a limitation of spherical splines that produces Gibbs ringing and spurious activations in unmeasured regions), the approach ensures spatial specificity whilst enabling rigorous statistical inference across the measurement domain.

#### 2.4.2 Physiological preprocessing

R-peaks were identified from the ECG signal using the Pan-Tompkins algorithm (Pan and Tompkins, 1985). Instantaneous heart rate was derived from the inter-beat interval series and interpolated onto a uniform time grid using shape-preserving piecewise cubic interpolation. Both the heart rate and GSR signals were subsequently downsampled to 1 Hz and aligned to the fNIRS time series via synchronization timestamps recorded at acquisition, with resampling performed using modified Akima interpolation (Akima, 1970).

### 2.5 Vividness ratings analysis

Pre-test questionnaire scores and post-test vividness ratings were examined to validate the experimental stimuli and to establish the basis for including imagery ability as a covariate in the neuroimaging GLM. Pearson's *r* was used throughout. **Pre-test Questionnaires:** VVIQ and VOIQ scores were reverse-scored so that higher scores reflect greater vividness and totalled (maximum 80 per modality). Scores were correlated across modalities to examine whether individuals with stronger imagery capacity in one sensory modality exhibit similarly stronger capacity in the other. **Post-test Vividness Ratings:** Participants rated imagery vividness for each experimental condition on a 5-point scale (1 = low vividness, 5 = high vividness), yielding total scores out of 35 per condition. Correlations between pre-test questionnaire scores and post-test vividness ratings were examined to assess whether the stimuli successfully elicited imagery consistent with participants' known imagery ability. Pre- and post-test scores were converted to proportions of maximum possible scores prior to correlation to permit direct comparison across instruments.

### 2.6 fNIRS analysis

#### 2.6.1 First-level GLM

For each subject, we constructed a first-level general linear model (Figure 4B, left panel) wherein each event type was modeled using a boxcar function spanning the block duration, convolved with the canonical haemodynamic response function and its temporal derivative. The temporal derivative was included to capture latency variability in the haemodynamic response across scalp locations and participants, which represents the dominant source of HRF variance (Handwerker et al., 2004); the dispersion derivative was omitted to preserve statistical power in the absence of a strong principled justification for its inclusion. To control for systemic physiological influences, we included regressors for heart rate [convolved with a cardiac response function; de la Cruz et al. (2017)] and tonic skin conductance. While we recognize that physiological regressors do not perform as well as short-separation channel regression (not available with our current setup), they are still a principled approach and have been shown to be more reliable than spatial decomposition methods (Yücel et al., 2021). The physiological regressors were subjected to Gram-Schmidt orthogonalisation against the experimental regressors prior to inclusion, ensuring they captured only variance unexplained by the experimental design and preventing inappropriate removal of task-related signal. We applied a high-pass filter (cutoff 128s) to remove slow drifts and implemented prewhitening using an autoregressive AR(1) model with coefficient 0.4, chosen to account for the higher temporal autocorrelation in fNIRS data compared to fMRI (Huppert, 2016). Experimental regressors were concatenated across the three runs. Nuisance regressors, including physiological signals and polynomial drift terms, were modeled separately for each run within the same GLM, such that slow signal drifts and physiological fluctuations specific to each run were accounted for independently without influencing the estimation of experimental effects. Following parameter estimation, we constructed three contrasts (main effect of Imagery Modality, main effect of Representation Type, and their interaction) for second-level analysis.

#### 2.6.2 Second-level GLM

For each first-level contrast, we constructed a second-level one-sample t-test model for group-level inference (Figure 4B right panel). Analyses were conducted separately for HbO, HbR, and CBSI. Participants' Vividness of Visual Imagery Questionnaire (VVIQ) and Vividness of Olfactory Imagery Questionnaire (VOIQ) scores were included as covariates to partial out variance attributable to subjective imagery ability. We evaluated directional effects using one-tailed *t*-tests: for Imagery Modality, testing visual > olfactory and olfactory > visual; for Representation Type, testing words > colors and colors > words; and for their interaction, testing where the effect of Representation Type differed between modalities. Statistical inference used random field theory to control familywise error rate at *p* < 0.05 across the entire scalp mesh. Findings surviving FWE correction are reported as primary results. Effect sizes are reported as Cohen's *d*, estimated at peak vertices as $d=T/\sqrt{N}$. Exploratory activations at *p* < 0.001 and *p* < 0.05 uncorrected are additionally reported to provide a comprehensive characterization of the data consistent with the exploratory nature of this feasibility study, and to inform the design and power calculations of future confirmatory work in which such effects may be replicated.

#### 2.6.3 Scalp-to-brain projection and anatomical labeling

Brain projections are provided for interpretative purposes. Scalp-level statistical maps were projected onto a canonical pial surface mesh using nearest-neighbor interpolation. Each vertex on the scalp mesh was mapped to its nearest vertex on the brain surface, with the corresponding statistical value assigned to that brain vertex. For tabulated peak statistics, scalp peak coordinates were individually mapped to their nearest pial surface location, and anatomical labels were assigned using the Neuromorphometrics cortical atlas (Neuromorphometrics, 2012). The nearest electrode position was identified for each scalp peak using Euclidean distance to an SPM-based extended electrode template, which provides 10-10 positions and intermediate locations (h).

#### 2.6.4 *Post-hoc* exploratory analysis: chromophore-specific activations and deactivations

Following the primary factorial analyses, a post-hoc exploratory analysis was conducted to characterize chromophore-specific haemodynamic response patterns within individual experimental conditions. This analysis was motivated by two considerations. First, the primary contrasts revealed discordant patterns across chromophores, with weak or absent effects in CBSI despite detectable signals in individual chromophores. Second, CBSI operates on the assumption that neural activation produces a reliable negative correlation between HbO and HbR (Cui et al., 2010). Whilst this assumption holds under standard stimulus-driven experimental conditions, the relationship between HbO and HbR is sensitive to the systemic physiological state of the participant, including variations in parameters such as partial pressure of carbon dioxide and resting cerebral blood flow, which can alter the expected chromophore correlation structure (Caldwell et al., 2016). In paradigms involving top-down, internally generated processes such as mental imagery, where task-related deactivations may produce haemodynamic dynamics that diverge from conventional stimulus-driven activation, the validity of CBSI assumptions warrants careful consideration. Whilst CBSI remains the appropriate convention for improving signal stability and is retained as a primary measure, the present post-hoc analysis examines individual chromophores independently to provide a more complete characterization of the haemodynamic response. Each experimental condition was examined against the implicit baseline separately for activations and deactivations, using a liberal statistical threshold (*p* < 0.001 uncorrected, minimum cluster extent *k*_*E*_ = 5 vertices). Findings are reported as descriptive and exploratory, intended to characterize chromophore-specific haemodynamic response patterns rather than to draw inferential conclusions.

## 3 Results

### 3.1 Vividness ratingsl

Self-reported measures of imagery vividness were analyzed to characterize individual differences in imagery ability and subjective vividness across experimental conditions. Trait imagery capacity was assessed using standardized questionnaires (VVIQ and VOIQ), providing baseline measures of visual and olfactory imagery vividness. Participants also provided vividness ratings for each of the four experimental conditions (visual and olfactory imagery evoked by verbal cues or color patterns), enabling examination of how cue type influences subjective imagery quality across modalities.

#### 3.1.1 Vividness questionnaires

The majority of participants (*N* = 25) reported greater vividness of visual imagery (55.09 ± 13.46) than olfactory imagery (48.77 ± 12.81), consistent with the general population trend that visual imagery is typically more vivid and accessible than imagery in other sensory modalities. Vividness scores were significantly correlated across visual and olfactory domains (*r*(34) = 0.5021; *p* = 0.0021), suggesting that individuals with strong imagery capacity in one modality tend to exhibit stronger imagery across modalities.

#### 3.1.2 Stimuli vividness ratings

**Figure 6:**
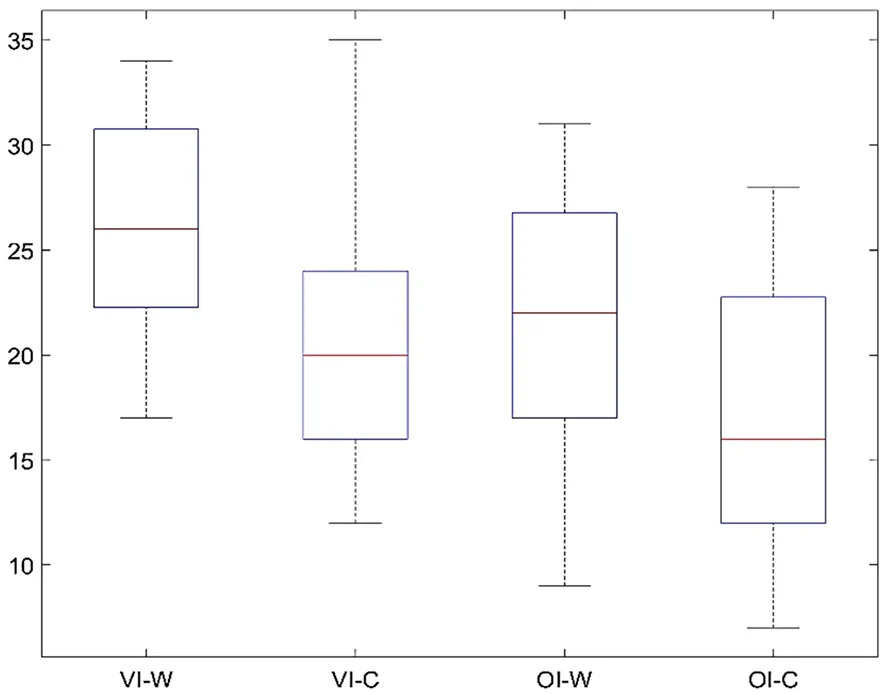
Distribution of imagery vividness ratings across experimental conditions. Boxplots show participants' self-reported vividness scores (out of 35) for each condition: visual imagery to words (VI-W), visual imagery to colors (VI-C), olfactory imagery to words (OI-W), and olfactory imagery to colors (OI-C). Box boundaries represent the interquartile range (25th–75th percentiles), the horizontal line within each box indicates the median, and whiskers extend to the most extreme data points within 1.5 times the interquartile range. Participants reported highest vividness for visual imagery evoked by words, with notably lower ratings for color-evoked imagery across both modalities. The overall pattern demonstrates greater vividness for word-based stimuli compared to color patches, and higher ratings for visual compared to olfactory imagery.

**Figure 7:**
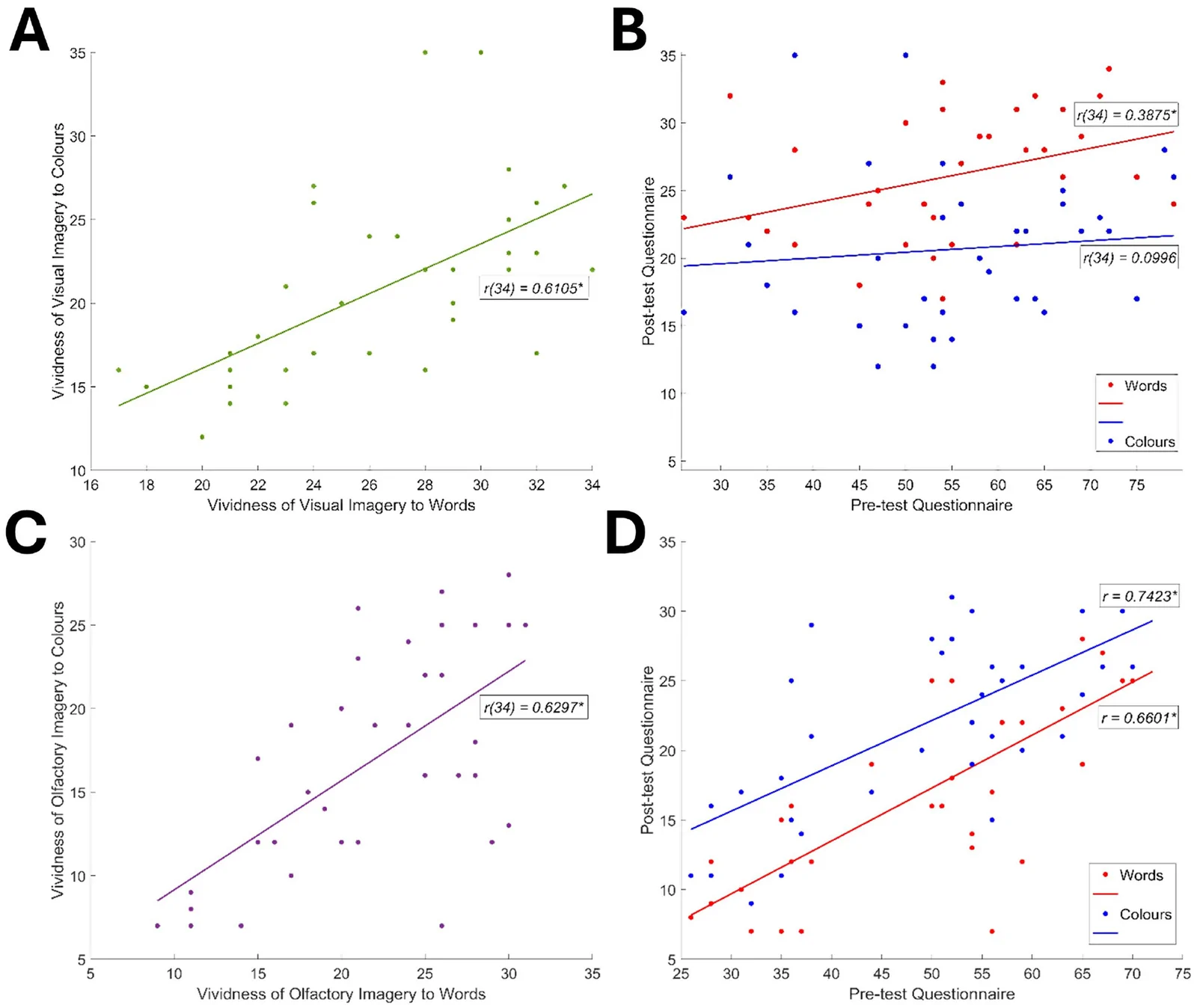
Correlations between imagery vividness ratings and questionnaire scores. **(A)** Scatter plot of participants' vividness ratings (out of 35) for visual imagery to words versus visual imagery to colors (green; *r*(34) = 0.6105, *p* < 0.001). Participants' vividness scores were highly positively correlated between these two experimental conditions. **(B)** Scatter plot of participants' post-test vividness ratings (out of 35) for visual imagery to words (red) and visual imagery to colors (blue) plotted against participants' pre-test VVIQ scores (out of 80). Vividness ratings for visual imagery to words were significantly correlated with VVIQ scores (*r*(34) = 0.3875, *p* = 0.0215), whilst vividness ratings for visual imagery to colors were not (*r*(34) = 0.0996, *p* = 0.5694). **(C)** Scatter plot of participants' vividness ratings (out of 35) for olfactory imagery to words versus olfactory imagery to colors (purple; *r*(34) = 0.6297, *p* < 0.001). Participants' vividness scores were highly positively correlated between these two experimental conditions. **(D)** Scatter plot of participants' post-test vividness ratings (out of 35) for olfactory imagery to words (red) and olfactory imagery to colors (blue) plotted against participants' pre-test VOIQ scores (out of 80). Vividness ratings for both olfactory imagery to words (*r*(34) = 0.6601, *p* < 0.001) and to colors (*r*(34) = 0.7423, *p* < 0.001) were strongly positively correlated with VOIQ scores.

Participants reported the highest vividness of mental imagery when forming visual mental images verbally-cued (26.11 ± 4.70) exceeding vividness ratings for visual imagery cued by colors (20.66 ± 5.73; Figure 6). This stimulus-type effect suggests that linguistic cues provide richer semantic scaffolding for imagery generation than perceptual features alone. Vividness ratings of verbal- and color-evoked visual imagery were significantly correlated (*r*(34) = 0.6105, *p* < 0.001; Figure 7A), indicating moderate consistency in individual visual imagery capacity across stimulus types. Crucially, vividness ratings of visual imagery verbally-cued (*r*(34) = 0.3875, *p* = 0.0215) were significantly correlated with VVIQ scores, whereas vividness ratings of visual imagery cued by colors (*r*(34) = 0.0996, *p* = 0.5694) were not (Figure 7B). This dissociation suggests that trait visual imagery ability, as measured by the VVIQ, preferentially predicts performance on semantically mediated imagery tasks rather than perceptually driven imagery, potentially reflecting the linguistic nature of the VVIQ itself.

The pattern for olfactory imagery closely mirrored visual imagery. Participants reported more vivid olfactory imagery to verbal cues (21.74 ± 6.32) than colors (16.83 ± 6.56; Figure 6), demonstrating that word superiority generalizes across modalities. Vividness ratings for olfactory imagery to words and colors were highly correlated (*r*(34) = 0.6297, *p* < 0.001; Figure 7C), indicating within-modality consistency. In contrast to visual imagery, both word-evoked (*r*(34) = 0.6601, *p* < 0.001) and color-evoked (*r*(34) = 0.7423, *p* < 0.001) olfactory imagery ratings strongly correlated with VOIQ scores (Figure 7D). These stronger correlations suggest olfactory imagery depends more uniformly on trait capacity regardless of stimulus type, potentially reflecting the more effortful nature of olfactory compared to visual imagery or the limited role of perceptual features in guiding olfactory imagery generation. The particularly strong correlation for color-evoked olfactory imagery (*r* = 0.74) indicates that individuals with strong olfactory imagery can generate scent representations even from abstract visual cues, a capacity that may be more challenging in the visual domain when semantic information is minimal.

### 3.2 fNIRS

Statistical parametric mapping of scalp haemodynamic responses revealed modality-specific and stimulus-dependent patterns of activation during mental imagery. Results are reported for correlation-based signal improvement (CBSI), oxyhaemoglobin (HbO), and deoxyhaemoglobin (HbR) separately, with each chromophore providing complementary information regarding cortical activation patterns. Concordance across chromophores supports the reliability of observed effects, whilst discordance highlights potential contributions from systemic physiological noise or differential sensitivity to neurovascular coupling.

#### 3.2.1 Main effect: imagery modality

**Figure 8:**
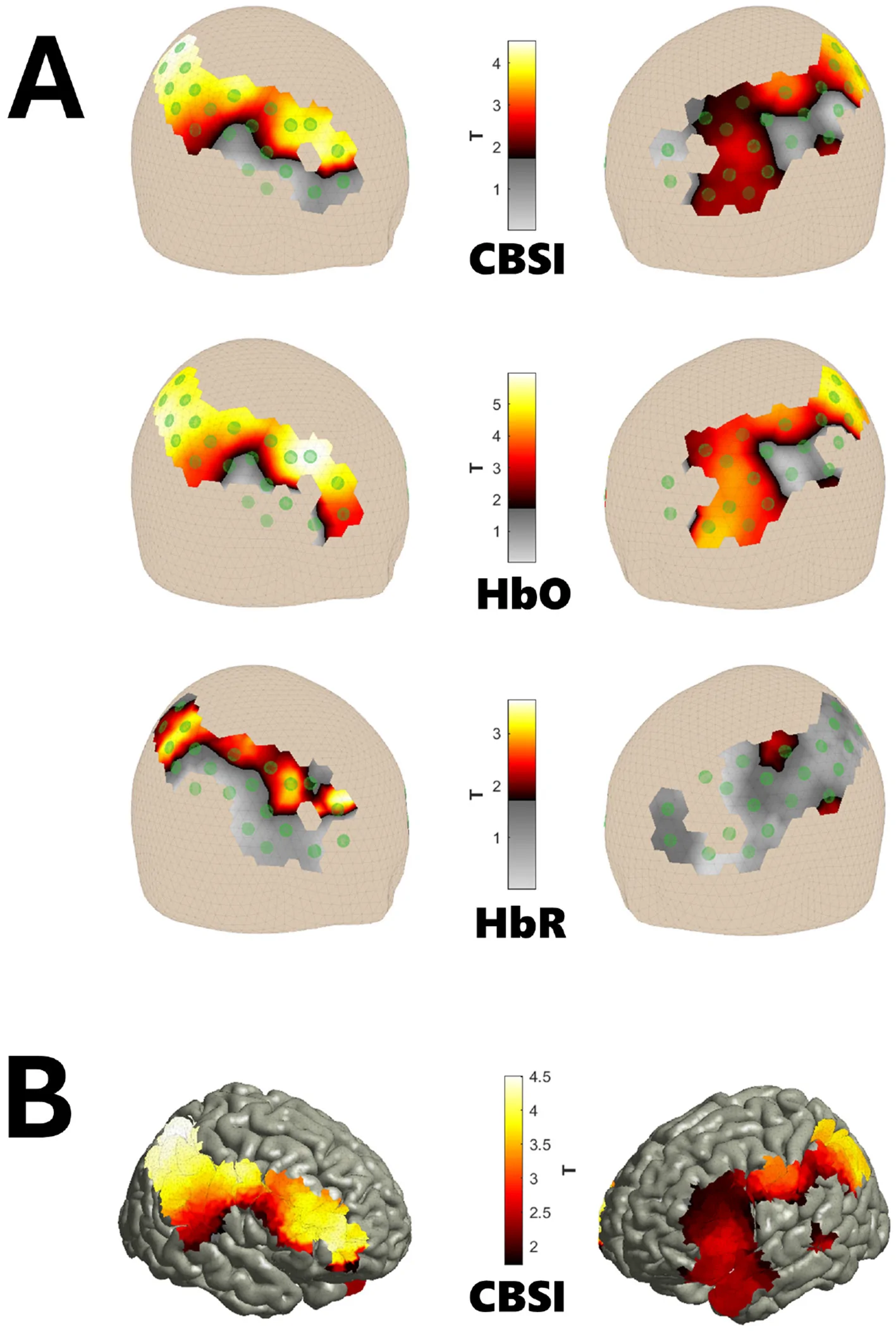
Main effect of imagery modality (visual > olfactory): statistical parametric maps showing scalp regions with greater activation during visual imagery compared to olfactory imagery. **(A)** Scalp-level results displayed on lateral views of the canonical scalp mesh for CBSI, HbO, and HbR signals separately, thresholded at *p* < 0.05 uncorrected for illustration purposes. Green circles indicate fNIRS channel locations. Color scales represent *T*-statistics. **(B)** CBSI results projected onto the pial surface for anatomical reference.

**Visual** **>** **olfactory imagery** Visual imagery elicited significantly greater cortical activation than olfactory imagery across multiple regions, with effects meeting familywise error correction thresholds evident across all chromophores (Table 1; Figure 8). CBSI analysis identified four distinct clusters surviving FWE correction (*p* < 0.05). The most prominent activation occurred in the right superior parietal lobule (peak MNI (brain): 41, -43, 74; *k*_*E*_ = 117; *p*_*FWE*_ = 0.003; *p*_*unc*_ = < 0.001; *T* = 4.51), a region implicated in spatial attention and imagery-based mental rotation (Kosslyn et al., 2001; Zacks, 2008). Additional CBSI effects were detected in right middle frontal gyrus (peak MNI (brain): 48, 46, 10; *k*_*E*_ = 49; *p*_*FWE*_ = 0.005; *p*_*unc*_ = < 0.001; *T* = 4.28), left angular gyrus (peak MNI (brain): -46, -73, 47; *k*_*E*_ = 51; *p*_*FWE*_ = 0.015; *p*_*unc*_ = < 0.001; *T* = 3.81), and left precentral gyrus (peak MNI (brain): -48, -7, 60; *k*_*E*_ = 2; *p*_*FWE*_ = 0.039; *p*_*unc*_ = < 0.001; *T* = 3.37). HbO measurements corroborated these CBSI findings whilst revealing somewhat broader spatial extent, particularly in prefrontal cortex. HbO analysis identified two large clusters: right middle frontal gyrus (peak MNI (brain): 47, 41, 29; *k*_*E*_ = 211; *p*_*FWE*_ < 0.001; *p*_*unc*_ = < 0.001; *T* = 5.95) and left superior parietal lobe (peak MNI (brain): -42, -51, 69; *k*_*E*_ = 180; *p*_*FWE*_ = 0.001; *p*_*unc*_ = < 0.001; *T* = 5.17). HbR analysis revealed a single small cluster in the right middle frontal gyrus (peak MNI (brain): 31, 57, 18; *k*_*E*_ = 1; *p*_*FWE*_ = 0.050; *p*_*unc*_ = 0.001; *T* = 3.64).

**Table 1:** Main effect of imagery modality (visual > olfactory): Peak statistics for clusters where visual imagery produced greater cortical activation than olfactory imagery.

	Peak-level						Scalp MNI (mm)				Projected Brain MNI (mm)			
Signal	*k*_E_	*p*_FWE_	*p*_unc_	*T*	(*Z*_E_)	*d*	x	y	z	10-10	x	y	z	Anatomy
CBSI *p*<0.05 (FWE-corrected)														
	117	0.003	< 0.001	4.51	3.86	0.82	45	-51	87	CCP2	41	-43	74	R SPL
	49	0.005	< 0.001	4.28	3.71	0.78	59	57	14	AFF6	48	46	10	R MFG
	51	0.015	< 0.001	3.81	3.38	0.70	-59	-79	53	CPP3	-46	-73	47	L AnG
	2	0.039	0.001	3.37	3.05	0.62	-60	-4	67	FCC3	-48	-7	60	L PrG
HbO *p*<0.05 (FWE-corrected)														
	211	< 0.001	< 0.001	5.95	4.72	1.09	57	50	34	F6h	47	41	29	R MFG
	180	0.001	< 0.001	5.17	4.27	0.94	-55	-55	78	CP3h	-42	-51	69	L SPL
HbR *p*<0.05 (FWE-corrected)														
	1	0.050	0.001	3.64	3.26	0.66	39	74	22	AF6h	31	57	18	R MFG

Results are reported separately for CBSI, HbO, and HbR signals. All clusters survived familywise error correction (p < 0.05). Peak-level statistics include cluster extent (*k*_E_), FWE-corrected *p*-value (*p*_FWE_), uncorrected *p*-value (*p*_unc_), *T*-statistic, and equivalent *Z*-score (*Z*_E_). Cohen's *d* (*n* = 30) is also reported for each peak, calculated as *d* = *T*/$\sqrt{n}$. Scalp MNI coordinates denote peak vertex locations on the canonical scalp mesh with corresponding extended 10-10 electrode positions. Projected brain MNI coordinates and anatomical labels identify the nearest cortical location. Anatomical abbreviations: R, right; L, left; SPL, superior parietal lobule; MFG, middle frontal gyrus; AnG, angular gyrus; PrG, precentral gyrus.

**Figure 9:**
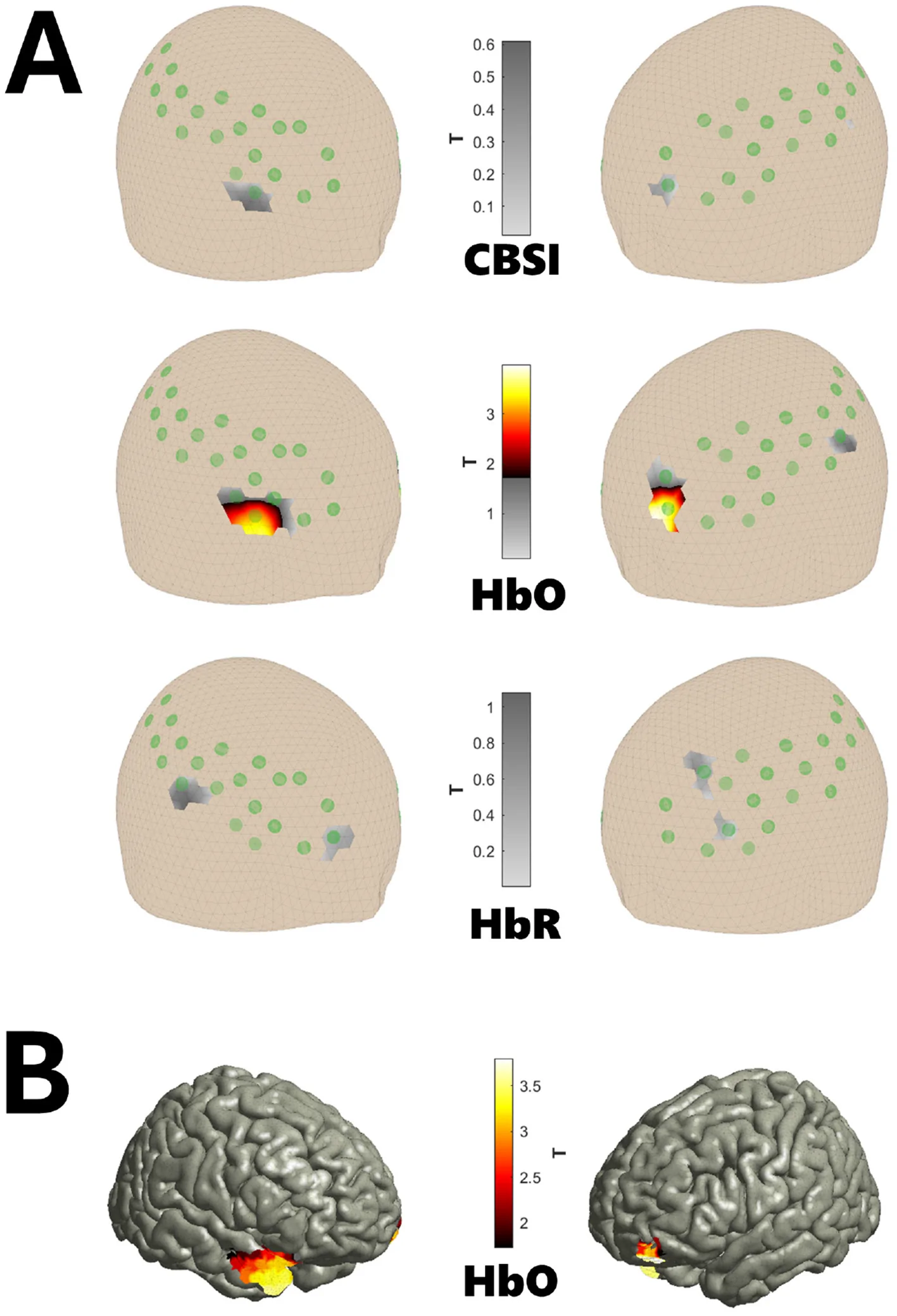
Main effect of imagery modality (olfaction > visual) statistical parametric maps showing scalp regions with greater activation during olfactory imagery compared to visual imagery. **(A)** Scalp-level results displayed on lateral views of the canonical scalp mesh for CBSI, HbO, and HbR signals separately, thresholded at *p* < 0.05 uncorrected for illustration purposes. Green circles indicate fNIRS channel locations. Color scales represent *T*-statistics. Note that only HbO revealed significant clusters surviving FWE correction. **(B)** HbO results projected onto the pial surface for anatomical reference, showing bilateral frontopolar activation.

**Olfactory** **>** **visual** Olfactory imagery produced significantly greater activation than visual imagery, though this effect was detected exclusively in HbO measurements (Table 2; Figure 9). Two small clusters survived FWE correction: left anterior orbital gyrus (peak MNI (brain): -27, 53, -5; *k*_*E*_ = 6; *p*_*FWE*_ = 0.011; *p*_*unc*_ = < 0.001; *T* = 3.98) and right temporal pole (peak MNI (brain): 51, 14, -20; *k*_*E*_ = 5; *p*_*FWE*_ = 0.037; *p*_*unc*_ = 0.001; *T* = 3.60). Neither CBSI nor HbR analyses revealed significant effects for this contrast, indicating that these findings should be interpreted with appropriate caution. As such, the detection of olfactory-preferential activation exclusively in HbO suggests potential physiological confounds that the CBSI correction procedure may have successfully attenuated. However, the anatomical specificity of these findings corresponds plausibly with olfactory processing networks. Both clusters localize to frontopolar scalp locations, with the left anterior orbital gyrus detected directly and the right temporal pole scalp location potentially reflecting right orbitofrontal activity. The anatomical projection from scalp to deeper brain structures warrants caution, particularly for ventral and orbital regions where sulcal variability and measurement depth introduce spatial uncertainty. Taking the brain projections as localizing to bilateral orbitofrontal cortex, these regions represent secondary olfactory cortical areas consistently implicated in odor processing and olfactory imagery in fMRI studies (Rolls et al., 1996). Nevertheless, whilst the bilateral frontopolar scalp activation surviving FWE correction suggests cortical involvement in olfactory imagery, the detection exclusively in HbO indicates these effects are either too weak to survive CBSI correction or potentially question the suitability of CBSI for mental imagery studies (see Discussion).

**Table 2:** Main effect of imagery modality (olfactory > visual): Peak statistics for clusters where olfactory imagery produced greater cortical activation than visual imagery.

	Peak-level						Scalp MNI (mm)				Projected Brain MNI (mm)			
Signal	*k*_E_	*p*_FWE_	*p*_unc_	*T*	(*Z*_E_)	*d*	x	y	z	10-10	x	y	z	Anatomy
HbO *p*<0.05(FWE-corrected)														
	6	0.011	< 0.001	3.98	3.50	0.73	-35	79	-16	AFp9h	-27	53	-5	L AOrG
	5	0.037	0.001	3.60	3.23	0.66	74	30	-26	FFT8	51	14	-20	R TMP

Results are reported for HbO signal only; neither CBSI nor HbR analyses revealed significant effects for this contrast. All clusters survived familywise error correction (p < 0.05). Peak-level statistics include cluster extent (*k*_E_), FWE-corrected *p*-value (*p*_FWE_), uncorrected *p*-value (*p*_unc_), *T*-statistic, and equivalent *Z*-score (*Z*_E_). Cohen's *d* (*n* = 30) is also reported for each peak, calculated as *d* = *T*/$\sqrt{n}$. Scalp MNI coordinates denote peak vertex locations on the canonical scalp mesh with corresponding 10-10 electrode positions. Projected brain MNI coordinates and anatomical labels identify the nearest cortical location. Anatomical abbreviations: L, left; R, right; AOrG, anterior orbital gyrus; TMP, temporal pole.

#### 3.2.2 Main effect: representation type

**Figure 10:**
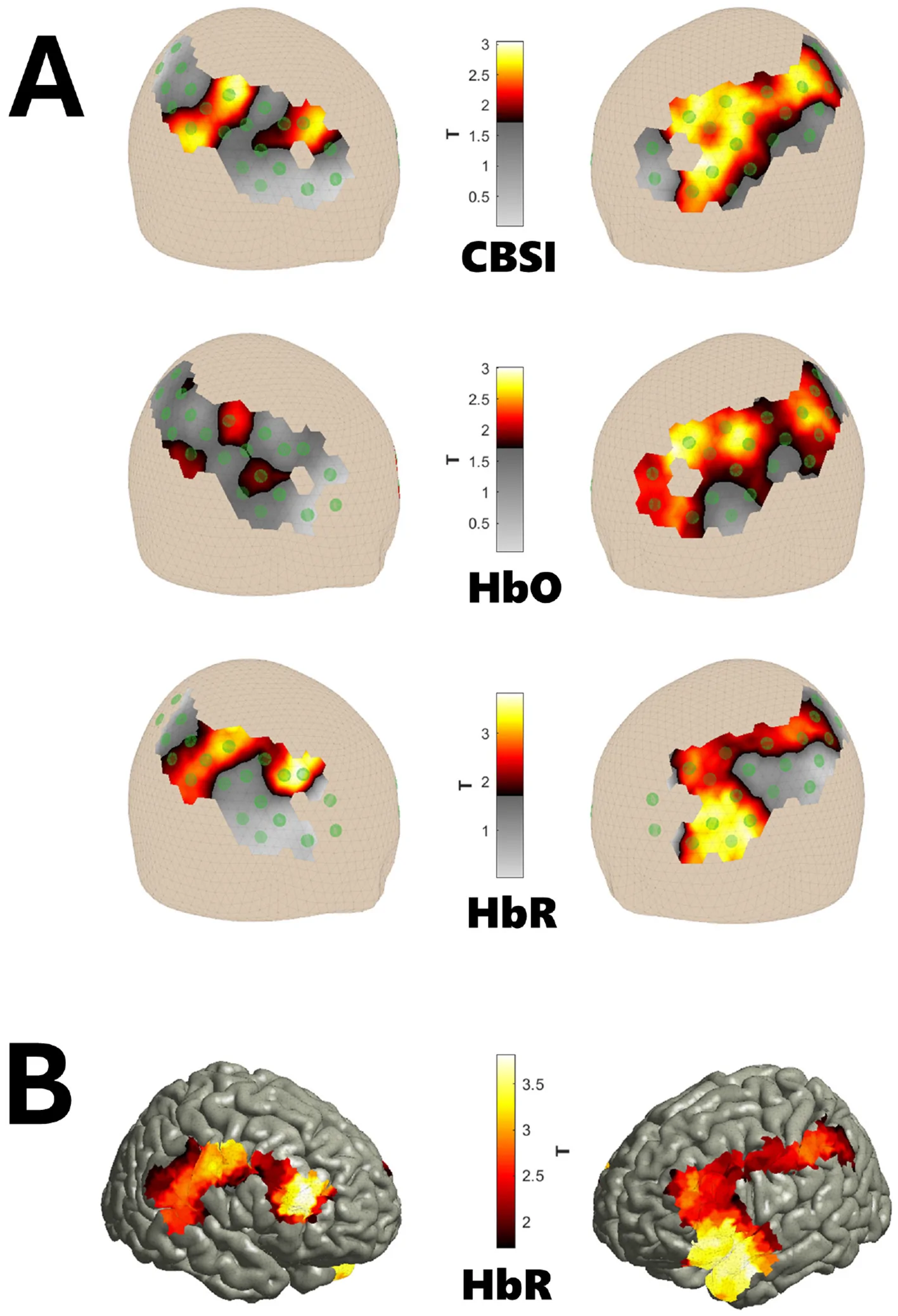
Main effect of representation type: (verbal > color pattern): statistical parametric maps showing scalp regions with greater activation during verbal cue-evoked imagery compared to color pattern-evoked imagery. **(A)** Scalp-level results displayed on lateral views of the canonical scalp mesh for CBSI, HbO, and HbR signals separately, maps are thresholded at *p* < 0.05 uncorrected for illustration purposes. Green circles indicate fNIRS channel locations. Color scales represent *T*-statistics. Note the spatial coherence across chromophores in bilateral frontal and temporal regions. **(B)** HbR results projected onto the pial surface for anatomical reference, showing the two small clusters that survived FWE correction in right middle frontal gyrus and left superior temporal gyrus.

**Verbal** **>** **color pattern:** Mental imagery evoked by verbal cues produced greater activation than color pattern-evoked imagery, with spatially coherent effects detected across chromophores, though these predominantly failed to reach FWE-corrected significance thresholds (Table 3; Figure 10). CBSI analysis revealed three clusters approaching FWE significance: left triangular part of the inferior frontal gyrus (peak MNI (brain): -51, 37, 7; *k*_*E*_ = 224; *p*_*FWE*_ = 0.097; *p*_*unc*_ = 0.003; *T* = 3.04), right precentral gyrus (peak MNI (brain): 59, 0, 49; *k*_*E*_ = 80; *p*_*FWE*_ = 0.115; *p*_*unc*_ = 0.003; *T* = 2.95), and right middle frontal gyrus (peak MNI (brain): 39, 49, 28; *k*_*E*_ = 37; *p*_*FWE*_ = 0.158; *p*_*unc*_ = 0.005; *T* = 2.78). HbO measurements demonstrated a consistent spatial pattern, with the strongest effect in the left middle frontal gyrus (peak MNI (brain): -37, 46, 30; *k*_*E*_ = 254; *p*_*FWE*_ = 0.095; *p*_*unc*_ = 0.003; *T* = 3.01), approaching FWE significance. Additional HbO clusters in right precentral gyrus (peak MNI (brain): 59, 0, 49; *k*_*E*_ = 44; *p*_*FWE*_ = 0.331; *p*_*unc*_ = 0.016; *T* = 2.26), right superior temporal gyrus (peak MNI (brain): 71, -29, 10; *k*_*E*_ = 20; *p*_*FWE*_ = 0.449; *p*_*unc*_ = 0.026; *T* = 2.02), and right supramarginal gyrus (peak MNI (brain): 52, -49, 63; *k*_*E*_ = 5; *p*_*FWE*_ = 0.582; *p*_*unc*_ = 0.045; *T* = 1.75) extended this bilateral frontal-temporal pattern, though with progressively weaker statistical support. HbR analysis identified two small clusters that survived FWE correction: right middle frontal gyrus (peak MNI (brain): 46, 41, 31; *k*_*E*_ = 5; *p*_*FWE*_ = 0.030; *p*_*unc*_ = < 0.001; *T* = 3.82) and left superior temporal gyrus (peak MNI (brain): -59, 1, -12; *k*_*E*_ = 2; *p*_*FWE*_ = 0.035; *p*_*unc*_ = < 0.001; *T* = 3.76). Crucially, these HbR findings overlap spatially with the trend-level CBSI and HbO effects in bilateral frontal and temporal regions, suggesting word-preferential activation that approaches but generally fails to reach conventional significance thresholds.

**Table 3:** Main effect of representation type: (verbal > color pattern): Peak statistics for clusters where verbal cue-evoked imagery produced greater cortical activation than color pattern-evoked imagery.

Peak-level						Scalp MNI (mm)				Projected Brain MNI (mm)				
Signal	*k*_E_	*p*_FWE_	*p*_unc_	*T*	(*Z*_E_)	*d*	x	y	z	10–20	x	y	z	Anatomy
CBSI *p*<0.05 (uncorrected)														
	224	0.097	0.003	3.04	2.79	0.56	-65	47	5	F7h	-51	37	7	L TrIFG
	80	0.115	0.003	2.95	2.72	0.54	71	2	53	FCC4	59	0	49	R PrG
	37	0.158	0.005	2.78	2.58	0.51	48	61	34	AFF4	39	49	28	R MFG
HbO *p*<0.05 (uncorrected)														
	254	0.095	0.003	3.01	2.77	0.55	-48	58	34	AFF5h	-37	46	30	L MFG
	44	0.331	0.016	2.26	2.15	0.41	71	2	53	FCC4	59	0	49	R PrG
	20	0.449	0.026	2.02	1.94	0.37	85	-24	14	C6	71	-29	10	R STG
	5	0.582	0.045	1.75	1.70	0.32	61	-50	72	CCP4h	52	-49	63	R SMG
HbR *p*<0.05 (FWE-corrected)														
	5	0.030	< 0.001	3.82	3.39	0.70	57	46	39	F4	46	41	31	R MFG
	2	0.035	< 0.001	3.76	3.34	0.69	-77	9	-15	FT7	-59	1	-12	L STG

Results are reported separately for CBSI, HbO, and HbR signals. CBSI and HbO clusters did not survive FWE correction and are reported at *p* < 0.05 uncorrected; HbR clusters survived FWE correction (p < 0.05). Peak-level statistics include cluster extent (*k*_E_), FWE-corrected *p*-value (*p*_FWE_), uncorrected *p*-value (*p*_unc_), *T*-statistic, and equivalent *Z*-score (*Z*_E_). Cohen's *d* (*n* = 30) is also reported for each peak, calculated as *d* = *T*/$\sqrt{n}$. Scalp MNI coordinates denote peak vertex locations on the canonical scalp mesh with corresponding 10-10 electrode positions. Projected brain MNI coordinates and anatomical labels identify the nearest cortical location. Anatomical abbreviations: L, left; R, right; TrIFG, triangular part of inferior frontal gyrus; PrG, precentral gyrus; MFG, middle frontal gyrus; STG, superior temporal gyrus; SMG, supramarginal gyrus.

The spatial coherence across chromophores, despite variable statistical thresholds, supports the interpretation that verbal cues engage bilateral frontal and temporal networks more strongly than color patches. The anatomical distribution corresponds plausibly with semantic processing, working memory, and language networks. Left inferior frontal gyrus supports semantic retrieval and lexical processing (Thompson-Schill et al., 1997), whilst bilateral frontal activations reflect executive control and working memory processes required for constructing mental images from linguistic descriptions (Emch et al., 2019). The temporal activations may reflect multimodal semantic associations supporting imagery construction (Binder et al., 2009). The predominantly trend-level nature of these findings (requiring liberal *p* < 0.05 uncorrected thresholds) likely reflects the modest effect sizes associated with stimulus-type differences. Nevertheless, the spatial consistency across chromophores, combined with the vividness ratings that words elicited more vivid imagery than colors, suggests these cortical patterns represent, albeit weak, neural signatures of semantic scaffolding in mental imagery construction.

**Color Pattern** **>** **Verbal:** No significant activation was detected for the reverse contrast (color pattern greater than verbal cues) in any chromophore, including at liberal uncorrected thresholds. This null finding aligns with vividness ratings that color patches consistently evoked less vivid imagery than verbal cues across both visual and olfactory modalities. The absence of color-preferential cortical activation suggests that the perceptual processing demands of color patches do not substantially exceed those of word reading within the cortical regions accessible to fNIRS, or alternatively, that any such processing occurs in regions outside the measurement domain.

#### 3.2.3 Interaction: imagery modality × representation type

Analysis of the interaction between imagery modality and representation type revealed no significant effects in any chromophore even at liberal thresholds. This null finding indicates that the effect of stimulus type (words versus color patches) on cortical activation did not differ significantly between visual and olfactory imagery conditions. The absence of interaction effects suggests that verbal and perceptual cueing mechanisms operate similarly across modalities within the cortical regions accessible to fNIRS, consistent with domain-general semantic and executive control processes supporting mental imagery regardless of sensory modality.

#### 3.2.4 *Post-hoc*: chromophore-specific patterns in activations and deactivations

**Figure 11:**
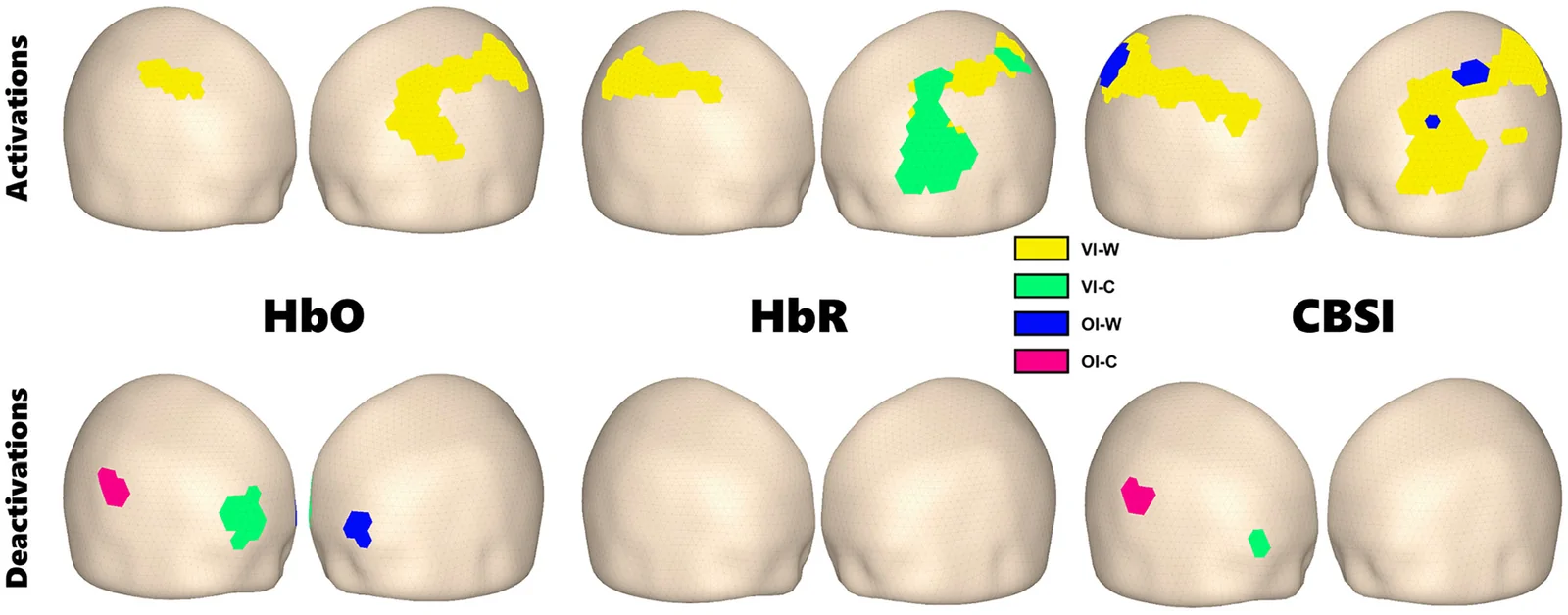
Chromophore-specific patterns of activations and deactivations. Scalp-level haemodynamic responses for each experimental condition were examined independently against an implicit baseline, displayed on lateral views of the canonical scalp mesh. Activations (top row) and deactivations (bottom row) are shown separately for HbO, HbR, and CBSI. All maps are thresholded at *p* < 0.001 uncorrected with minimum cluster extent *k*_E_ = 5 voxels. Color coding indicates experimental conditions: VI-W (yellow) = visual imagery to words; VI-C (green) = visual imagery to colors; OI-W (blue) = olfactory imagery to words; OI-C (magenta) = olfactory imagery to colors. Note the high concordance between HbO and HbR for VI-W activations, which survived CBSI correction. Deactivations were detected exclusively in HbO, with only color-cue conditions (VI-C and OI-C) surviving CBSI correction.

Given the discordant chromophore patterns observed in the primary contrasts, a post-hoc exploratory analysis was conducted to characterize activations and deactivations across individual chromophores for each experimental condition (Figure 11).

**Activations:** Visual imagery to words produced the most robust pattern, with high concordance between HbO and HbR. Both chromophores revealed diffuse bilateral frontoparietal activations with substantial spatial overlap. This concordance survived CBSI correction, yielding clear bilateral frontoparietal activations in the composite signal. Visual imagery to colors produced substantial left frontotemporal activation in HbR that was conspicuously absent in HbO, resulting in loss of this activation in CBSI. Olfactory imagery to words and colors showed absent activation in individual chromophores, though small patches of activation emerged in CBSI for olfactory imagery to words, despite the absence of detectable effects in HbO or HbR independently.

**Deactivations:** HbO revealed spatially distinct deactivation patterns for three conditions: visual imagery to colors (right frontooccipital), olfactory imagery to words (left frontooccipital), and olfactory imagery to colors (right parietotemporal). HbR showed no deactivations for any condition. Of the HbO deactivations, only those associated with color-pattern cues (both visual and olfactory) survived CBSI correction, whilst word-cue deactivations were lost.

## 4 Discussion

This study evaluated the feasibility of using fNIRS to detect cortical activations associated with olfactory and visual mental imagery evoked through verbal and perceptual cueing, with the central question being whether fNIRS can detect any cortical signature of olfactory imagery in higher-order regions accessible to the technique, despite its inability to reach primary olfactory structures in piriform cortex and medial temporal lobe.

Our results provide qualified support for this feasibility, whilst also revealing a set of findings that warrant careful interpretive consideration. Visual imagery to words produced the most robust bilateral frontoparietal activations, evident across all chromophores with high concordance between HbO and HbR, providing internal validation that the paradigm and array are sensitive to imagery-related cortical activity in accessible regions. Crucially, olfactory imagery produced detectable cortical activation in orbitofrontal and inferior frontal regions surviving familywise error correction, demonstrating the feasibility of fNIRS for studying olfactory imagery, though olfactory-preferential activation was detected exclusively in HbO measurements without corresponding concordance in HbR. No significant interaction between imagery modality and cue type was observed, suggesting that verbal and perceptual cueing mechanisms operate similarly across modalities within the cortical regions accessible to fNIRS. The post-hoc analysis further revealed that visual imagery to words was the only condition producing chromophore-concordant activations that survived CBSI correction, whilst all other conditions showed varying degrees of chromophore discordance. Deactivation patterns added a further layer of complexity, with HbO detecting spatially distinct deactivations for three conditions whilst HbR showed none, and only color-cue deactivations surviving CBSI correction. Together these findings raise important questions regarding chromophore coupling dynamics during mental imagery and the suitability of CBSI-corrected signals for endogenously driven cognitive processes, which are addressed in turn in the sections that follow. Whilst the presence of detectable signals is encouraging and speaks to the feasibility of this approach, the failure of many effects to survive CBSI correction leaves open the question of whether this reflects the modest nature of cortical activations associated with mental imagery, or whether the assumptions underlying CBSI are not fully appropriate for endogenously driven cognitive paradigms of this kind.

### 4.1 Mental imagery

The anatomical distribution of visual imagery activation aligns closely with established networks identified through fMRI investigations. Superior parietal activation corresponds to regions consistently implicated in spatial attention, mental rotation, and visuospatial transformations (Kosslyn et al., 2001; Zacks, 2008). Middle frontal gyrus contributions likely reflect executive control processes required for maintaining and manipulating visual mental images (Curtis et al., 2004), whilst angular gyrus activation may support semantic retrieval and multimodal integration during imagery construction (Seghier, 2013). The correspondence between these fNIRS-detected activations and established visual imagery networks (Fulford et al., 2018; Zvyagintsev et al., 2013) demonstrates that the technique provides sufficient sensitivity to detect imagery-related cortical activity in accessible regions, validating its potential for investigating mental imagery processes in contexts where fMRI remains impractical.

The central question motivating this investigation concerned whether fNIRS could detect cortical activations associated with olfactory imagery, given that primary olfactory structures lie beyond the technique's measurement depth. The findings provide highly suggestive but not definitive support for this possibility. The olfactory activation in orbitofrontal and inferior frontal regions aligns with prior neuroimaging evidence as plausible sites for detecting accessible olfactory-related activity (Rolls, 2004; Rolls et al., 1996; Li et al., 2010). Orbitofrontal cortex receives direct projections from piriform cortex and is consistently implicated in higher-order olfactory processing, including odor identification, hedonic evaluation, and cross-modal integration (Kringelbach, 2005). The detection of olfactory-preferential activation in this region represents an encouraging proof of concept. However, several features of the olfactory imagery findings warrant interpretive caution. Specifically, effects emerged exclusively in HbO measurements without corresponding concordance in HbR, a pattern that complicates attribution to neuronal activity. When contrasted with the robust, spatially extensive, and chromophore-concordant visual imagery effects, the olfactory findings appear tentative rather than definitive. This tentative finding likely reflects the fundamental constraint that primary olfactory imagery networks reside predominantly in piriform cortex, amygdala, and hippocampus, structures at depths of 4 cm or greater from the scalp surface, far exceeding the approximately 1.5 cm penetration depth achieved with standard source-detector separations. However, the spatial specificity of this orbitofrontal activation, combined with sophisticated methodological controls for systemic influences and rigorous multiple comparison correction, lends confidence to this finding despite its limitation to HbO measurements alone.

Beyond the technical limitations of fNIRS, the challenge of designing olfactory imagery paradigms warrants consideration. The deep location of primary olfactory processing regions reflects the evolutionary development of this sense and provides a plausible explanation for why olfactory imagery exhibits less volitional control than visual imagery. This anatomical organization may explain why engaging in olfactory imagery volitionally recruits other sensory modalities. Neuroimaging studies support this account, revealing that imagining scents activates not only primary olfactory cortex but also visual processing regions, suggesting functional coupling during mental simulation (Djordjevic et al., 2005). Further, when participants engage in odor imagery, they frequently report spontaneous visual images of the odor source or associated contexts (Stevenson and Case, 2005), and individuals with stronger visual imagery abilities demonstrate enhanced olfactory imagery capacity (Djordjevic et al., 2004). The vividness ratings corroborate this relationship, with participants reporting significantly more vivid imagery during visual compared to olfactory conditions and vividness ratings across modalities being positively correlated, suggesting that olfactory imagery is inherently more effortful and less reliably engaged than visual imagery. This cross-modal coupling represents an important conceptual limitation of the imagery modality contrast employed here, as olfactory imagery likely recruited visual processing regions alongside olfactory-specific activation, meaning the olfactory greater than visual contrast was not a comparison between fully independent processes and its sensitivity to detecting olfactory-preferential cortical activity would have been correspondingly reduced. These considerations, combined with the inherently more effortful and variable nature of olfactory imagery, may collectively account for the modest effects observed. Intriguingly, rather than producing clear task-positive activations, olfactory imagery appears to be associated predominantly with suppression of activity in accessible cortical regions, a pattern that is explored further in the following section.

### 4.2 Mental imagery, deactivations & CBSI

A striking finding concerns the patterns of chromophore discordance observed across experimental conditions, wherein responses appear in one chromophore but not the other, or emerge only after CBSI correction, and deactivations were detected exclusively in HbO, whilst HbR showed none whatsoever. A conventional interpretation of these chromophore discordance patterns would be to substantially weaken the inferential status of the findings, and this must be given serious consideration alongside the broader limitations of the present study, which are addressed in full in the limitations section. However, it is equally important to note that the present study was subjected to a number of methodological controls that lend some confidence to the observed effects, with physiological nuisance regressors included in the GLM and statistical inference performed using random field theory-based familywise error correction. We therefore suggest that whilst the chromophore discordance warrants interpretive caution, there is sufficient methodological justification to take the observed effects seriously and to ask whether the discordance itself may reflect something meaningful about the suitability of CBSI assumptions in this domain, rather than simply indicating that the findings are artifactual.

The CBSI algorithm assumes a strong zero-lag negative correlation between HbO and HbR during neuronal activation, a relationship that is well established for externally driven sensory paradigms (Cui et al., 2010) but whose validity in the context of task-related deactivations and endogenously driven cognitive processes has not, to our knowledge, been systematically examined. Deactivations during mental imagery are well established in the fMRI literature, with mental imagery robustly suppressing task-irrelevant sensory processing (Amedi et al., 2005; Azulay et al., 2009; Zvyagintsev et al., 2013), and the spatially specific deactivations observed here are broadly consistent with this pattern. Whether the zero-lag anticorrelation assumed by CBSI holds during such suppressive haemodynamic responses, however, remains an open and important question.

We offer the following reasoning explicitly as a speculative hypothesis warranting future investigation. There are principled biological grounds for suspecting that the neurovascular coupling dynamics underlying mental imagery may differ from those assumed by CBSI. Brain regions central to mental imagery, particularly default mode network areas, exhibit elevated aerobic glycolysis wherein glucose consumption exceeds oxidative phosphorylation requirements (Vaishnavi et al., 2010; Raichle and Snyder, 2007; Buckner et al., 2008), a metabolic profile that may produce haemodynamic responses differing from the canonical coupling on which CBSI is predicated. Furthermore, mental imagery is characterized by predominant top-down alpha and beta oscillatory activity (Bastos et al., 2015; van Ede et al., 2014) rather than the metabolically demanding gamma oscillations associated with bottom-up stimulus-driven processing (Lachaux et al., 2007; Mukamel et al., 2005; Muthukumaraswamy, 2010), and these distinct electrophysiological signatures are known to map onto fundamentally different neurovascular processes. Taken together, these considerations provide a plausible biological basis for suspecting that the zero-lag anticorrelation assumed by CBSI may not fully characterize the haemodynamic signature of endogenously driven cognition, and we hope these observations motivate future work examining whether CBSI assumptions generalize appropriately across the full range of cognitive domains in which fNIRS is increasingly being applied.

### 4.3 Limitations

Several limitations of the present study warrant consideration. The most significant methodological concern is the absence of short-separation channel regression, which represents the current standard for isolating cortical from extracerebral haemodynamic contributions in fNIRS. This is of particular relevance to the orbitofrontal HbO findings, as frontopolar and orbitofrontal regions are susceptible to extracerebral contamination, and without short-channel regression, a contribution from non-neural sources cannot be definitively excluded. Whilst physiological nuisance regressors were included in the GLM to provide partial control for systemic influences, this does not fully substitute for short-separation measurements. Importantly, the present findings provide a basis for informing short-channel placement in future work, which can now be targeted to the orbitofrontal and inferior frontal regions where olfactory imagery effects were observed.

The reliance on HbO alone for the olfactory imagery findings is a related limitation. The absence of corresponding HbR effects means that chromophore concordance cannot be established for the olfactory imagery signal, and this must be considered when interpreting the strength of the feasibility claim. The modest sample size, whilst consistent with comparable fNIRS feasibility investigations, may have been insufficient to detect the full range of cortical activations associated with olfactory imagery, and the conceptual overlap between imagery modalities represents a further constraint on the interpretability of the modality contrast. The block design may average over variable cognitive states within blocks, and future event-related designs would improve sensitivity to transient imagery-related activation.

### 4.4 Future work

This study revealed discordant haemodynamic patterns across chromophores, with activations and deactivations manifesting differently in HbO and HbR, and in some cases emerging only following CBSI correction. Future work should treat chromophore (de-)coupling as a feature of interest rather than a nuisance, and in particular, examine the validity of CBSI assumptions in top-down, internally driven paradigms of this kind, where the expected negative correlation between HbO and HbR may not hold in the same way as in conventional stimulus-driven designs (Caldwell et al., 2016).

One promising avenue for addressing this is dynamic causal modeling (DCM), which provides a biophysical framework for directly estimating the temporal coupling between HbO and HbR rather than assuming a fixed relationship, thereby relaxing the strong assumptions inherent in CBSI (Friston et al., 2019; Zeidman et al., 2019). When applied to fNIRS data, DCM can disambiguate bottom-up from top-down neural message passing and their distinct haemodynamic consequences (Tak et al., 2015). Crucially, DCM can accommodate hidden sources, enabling inference about deep structures such as limbic regions that are not directly accessible to fNIRS, making it particularly well suited to investigations of olfactory imagery (David et al., 2011). These properties, combined with existing demonstrations of DCM's utility in supporting predictive processing accounts of mental imagery (Dijkstra et al., 2017) and resolving olfactory message passing using non-invasive neuroimaging (Rho et al., 2021), suggest that DCM for fNIRS represents a principled and promising framework for future investigations of olfactory imagery neurovascular dynamics.

### 4.5 Conclusions

This study provides a first account of characterizing olfactory imagery using fNIRS. The technique demonstrated robust sensitivity to visual imagery in accessible frontoparietal networks, validating the experimental and analytical framework, whilst olfactory imagery produced tentative, HbO-only activation in orbitofrontal regions where theory predicts accessible olfactory-related activity. The absence of corresponding HbR effects means these findings should be interpreted cautiously and treated as preliminary rather than definitive evidence of neural activation. The fundamental constraint remains the depth limitation, placing primary olfactory structures beyond optical reach, positioning fNIRS as best suited for investigating higher-order cognitive contributions to olfactory processing, including semantic access, cross-modal integration, executive control, and hedonic evaluation, rather than primary sensory imagery itself. For researchers and practitioners in olfactory cognition, consumer neuroscience, and food science, this represents both limitation and opportunity: whilst the technique cannot characterize primary sensory olfactory processing, it offers a portable, naturalistic-compatible method for examining how olfactory expectations and experiences are shaped by cognitive, contextual, and evaluative processes in the frontal networks accessible to measurement. The methodological innovations introduced, including geodesic interpolation onto canonical scalp meshes, integration with SPM25 inference machinery, and orthogonalised physiological noise control, advance the analytical toolkit for fNIRS research beyond this specific application, and the tentative orbitofrontal effects observed suggest that hedonic and evaluative aspects of olfactory cognition may prove tractable targets for future fNIRS investigation.

## Data Availability

The datasets presented in this article are not readily available because the raw neuroimaging data underlying this study are not publicly available due to commercial confidentiality and proprietary restrictions. However, the processed data required to replicate the results are available from the corresponding author upon reasonable request and subject to a data-sharing agreement. Requests to access the datasets should be directed to I.Tachtsidis@metabolightltd.co.uk.
