# Supplementary material for: Shedding light on imagined scents: an fNIRS feasibility study of olfactory mental imagery and semantic cueing
Source: Front Neurosci. 2026 Aug 10;20:1825171. doi: 10.3389/fnins.2026.1825171 (PMC13500555; doi:10.3389/fnins.2026.1825171)
Supplement: Supplementary file 1 [file Data_Sheet_1.pdf]

Supplementary Material

Scalp Projection Procedure: Technical Specification and Code

Channel-level haemodynamic data were projected onto a canonical scalp surface mesh (2,562 vertices) prior to statistical parametric mapping using a custom procedure implemented within SPM. This procedure was developed to enable mass-univariate inference with random field theory-based familywise error correction, and represents the only custom component of the preprocessing pipeline. By mapping individual participant data onto a canonical mesh identical to that used for SPM M/EEG source reconstruction (Mattout et al., 2007), the procedure enables vertex-wise group statistics at corresponding scalp positions whilst accounting for individual variation in head geometry and optode placement.

The procedure comprises two stages: (1) geodesic Gaussian interpolation of channel data onto scalp mesh vertices, implemented in dot_interp_geodesic_gauss; and (2) application of a cosine-tapered boundary mask to suppress extrapolation artefacts at ROI edges, implemented within dot_glm_first_data. Both are described and listed in full below.

Stage 1: Geodesic Gaussian Interpolation

1.1 Mesh Specification

The canonical scalp mesh comprises 2,562 vertices with approximately equal spacing (mean edge length ~5 mm), satisfying the stationarity requirements for Gaussian random field theory. The canonical mesh enables vertex-wise group statistics at corresponding scalp positions despite individual variation in channel locations.

1.2 Channel-to-Vertex Anchor Mapping

Each fNIRS channel location in MNI space is mapped to its nearest scalp mesh vertex by minimising Euclidean distance. These nearest vertices serve as anchors. Channels sharing the same anchor vertex are averaged prior to interpolation.

1.3 Geodesic Distance Computation

The scalp mesh is represented as a weighted graph in which edges connect adjacent vertices with weights equal to Euclidean edge lengths in millimetres. Geodesic distances from all anchor vertices to all destination vertices are computed simultaneously using Dijkstra's algorithm via MATLAB's graph/distances function, preserving true surface distances over curved regions such as the temporal lobes.

1.4 Fixed-Width Gaussian Kernel Interpolation

For each destination vertex v, interpolation weights are computed as:

$$w(v, a) = exp(- d_{\gamma}(v, a)^2 / 2\sigma^2)$$

where $d_{Y}(v, a)$ is the geodesic distance from vertex v to anchor a, and σ = FWHM / √(8 ln 2) with FWHM = 30 mm. Weights are zeroed for distances exceeding 3σ (~38 mm) and row-normalised to ensure partition of unity:

$$\Sigma_a\ w(v, a) = 1 \quad \text{for each vertex } v$$

1.5 Vertex-Wise Signal Reconstruction

Vertex time series are computed via sparse matrix multiplication:

$$Y_{v}{}^{er}{}_{t}{}^{ex} = W \times Y^{anchors}$$

where W is the [M × N] sparse weight matrix and Y*anchors* is the [N × T] anchor time series matrix. Vertices outside the destination index are set to zero.

1.6 Code: dot_interp_geodesic_gauss.m

```
function [WY, W0, anchors, sigma_mm] = dot_interp_geodesic_gauss( ...
        scalp, xyz_vch, y, FWHM_mm, dest_idx, cutoff_sigma)
% Fixed-width geodesic Gaussian mapping from fNIRS channels to scalp vertices.
% Keeps a single spatial kernel width everywhere to respect SPM's
% approximately stationary smoothness assumption.
%
% Inputs
%   scalp.vertices : [nVert x 3] mm
%   scalp.faces    : [nFace x 3]
%   xyz_vch        : [nCh  x 3] channel locations on scalp (mm)
%   y              : [nSamp x nCh] data (time x channel)
%   FWHM_mm        : scalar Gaussian FWHM in mm
%   dest_idx       : indices or logical mask of destination vertices
%   cutoff_sigma   : optional. Zero weights for d > cutoff_sigma*sigma. Default 3.
%
% Outputs
%   WY        : [nVert x nSamp] vertexwise data
%   W0        : [M x N] sparse mapping matrix
%   anchors   : [N x 1] vertex indices used as channel anchors
%   sigma_mm  : Gaussian sigma in mm corresponding to FWHM_mm

    if nargin < 6 || isempty(cutoff_sigma), cutoff_sigma = 3; end
    V = double(scalp.vertices);
    F = double(scalp.faces);
    y = double(y);
    nVert = size(V,1);

    if nargin < 5 || isempty(dest_idx), dest_idx = (1:nVert).T; end
    if islogical(dest_idx), dest_idx = find(dest_idx); end
    dest_idx = dest_idx(:);
    M = numel(dest_idx);

    % convert FWHM to sigma
    sigma_mm = FWHM_mm / sqrt(8*log(2));
    cutoff_mm = cutoff_sigma * sigma_mm;

    % map channels to nearest scalp vertices (anchors)
```

```
    nCh = size(xyz_vch,1);
    xyz_vch = double(xyz_vch);
    anch = zeros(nCh,1);
    for k = 1:nCh
        [~, anch(k)] = min(vecnorm(V - xyz_vch(k,:), 2, 2));
    end
    [anchors, ~, ch2a] = unique(anch, 'stable');
    N = numel(anchors);

    % build mesh graph and compute multi-source geodesic distances
    E = [F(:,[1 2]); F(:,[2 3]); F(:,[3 1])];
    E = unique(sort(E,2),'rows');
    w = vecnorm(V(E(:,1),:) - V(E(:,2),:), 2, 2);
    G = graph(E(:,1), E(:,2), w);
    D = distances(G, anchors);   % [N x nVert] geodesic distances

    % assemble fixed-width Gaussian weights on destination vertices
    I = cell(M,1); J = cell(M,1); Val = cell(M,1);
    sig2 = sigma_mm^2;
    inv2sig2 = 1 / (2*sig2);

    for jj = 1:M
        v = dest_idx(jj);
        d = D(:, v);
        if isfinite(cutoff_mm)
            keep = d <= cutoff_mm;
        else
            keep = true(size(d));
        end
        if ~any(keep)
            [~, imin] = min(d);
            keep(imin) = true;
        end
        dk = d(keep);
        wk = exp(-(dk.^2) * inv2sig2);
        if ~any(wk), wk = ones(size(dk)); end
        wk = wk / sum(wk);   % partition of unity
        idx = find(keep);
        I{jj} = repmat(jj, numel(idx), 1);
        J{jj} = idx;
        Val{jj} = wk;
    end

    I = double(cell2mat(I)); J = double(cell2mat(J)); Val = double(cell2mat(Val));
    W0 = sparse(I, J, Val, M, N);   % [M x N], row sums == 1

    % aggregate channels sharing an anchor by averaging
    nSamp = size(y,1);
    Ya = zeros(nSamp, N);
    for a = 1:N
        Ya(:,a) = mean(y(:, ch2a == a), 2);
    end

    % apply mapping to surface
    WY = zeros(nVert, nSamp);
    WY(dest_idx, :) = W0 * Ya.T;   % [M x N]*[N x nSamp]
end
```

Stage 2: Cosine-Tapered Boundary Mask

2.1 ROI Definition

A region of interest is defined comprising all scalp mesh vertices within 20 mm (Euclidean distance) of any channel position in MNI space.

2.2 Boundary Taper

A cosine-tapered mask is applied to the interpolated vertex data to suppress extrapolation artefacts at ROI boundaries. For each vertex, the Euclidean distance to the nearest channel is computed. The mask is then:

$$m(v) = 1 \qquad \text{if } d(v) \leq 0.7r$$

$$m(v) = 0.5 + 0.5\cos(\pi\,(d(v) - 0.7r) / 0.3r) \qquad \text{if } 0.7r < d(v) < r$$

$$m(v) = 0 \qquad \text{if } d(v) \geq r$$

where r = 20 mm. The mask is applied element-wise to the interpolated vertex time series after interpolation.

2.3 Code: Boundary Masking (from dot_glm_first_data.m)

```
% --- Extract relevant section from dot_glm_first_data.m ---

% Define ROI vertices within radius r of any channel (via SPM)
r = 20;   % mm
[vert_in, xyz_in] = spm_fnirs_roivert(xyz_ch, r);

% Map each channel to nearest scalp vertex
for i = 1:nch
    xyz_vch(i,:) = spm_fnirs_vert_mindist(xyz_ch(i,:), scalp.vertices);
end

% Compute Euclidean distance from each vertex to nearest channel
dist_to_nearest = zeros(nvert, 1);
for i = 1:nvert
    dists = sqrt(sum(bsxfun(@minus, xyz_vch, scalp.vertices(i,:)).^2, 2));
    dist_to_nearest(i) = min(dists);
end

% Define valid region and cosine taper
max_dist    = r * 1.0;
valid_region = dist_to_nearest <= max_dist;
taper_start = r * 0.7;   % begin taper at 70% of radius
taper_end   = r * 1.0;   % zero weight at 100% of radius
taper_mask  = ones(nvert, 1);
taper_region = dist_to_nearest > taper_start;
taper_mask(taper_region) = 1 - (dist_to_nearest(taper_region) - taper_start) ...
                              / (taper_end - taper_start);
taper_mask(taper_mask < 0) = 0;
taper_mask(~valid_region)  = 0;
```

```
% Run geodesic Gaussian interpolation
FWHM_mm = 30;
[WY, W0, anchors, sigma_mm] = dot_interp_geodesic_gauss( ...
    scalp, xyz_vch, y, FWHM_mm, vert_in, 3);

% Apply cosine taper to interpolated vertex data
WY = WY .* repmat(taper_mask, 1, nsamp);
```
