# Supplementary material for: Shedding light on imagined scents: an fNIRS feasibility study of olfactory mental imagery and semantic cueing
Source: Front Neurosci. 2026 Aug 10;20:1825171. doi: 10.3389/fnins.2026.1825171 (PMC13500555; doi:10.3389/fnins.2026.1825171)
Supplement: Supplementary file 2 [file Data_Sheet_2.pdf]

| Patterns | Target odorant | hexa | R |
|---|---|---|---|
| 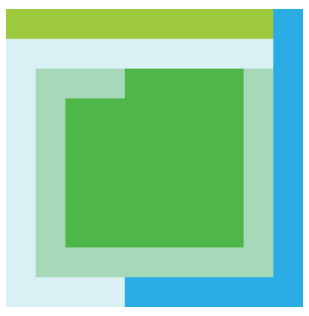 | Peppermint | #99FFCA | 153 |
| | | #4EB748 | 78 |
| | | #D3FFFF | 211 |
| | | #00B0F0 | 0 |
| | | #9ECA3C | 158 |
| 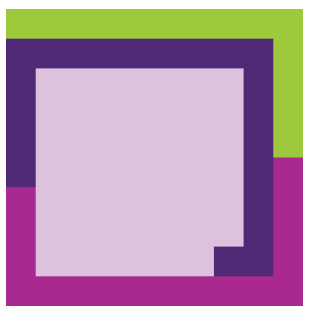 | Lavender | #9ECA3C | 158 |
| | | #E4C2F4 | 228 |
| | | #4F107A | 79 |
| | | #FF00E1 | 255 |
| 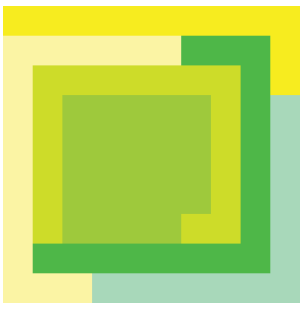 | Cucumber | #FFFFA1 | 255 |
| | | #FFFC02 | 255 |
| | | #BFDF1F | 191 |
| | | #9ECA3C | 158 |
| | | #4EB748 | 78 |
| | | #D3FFFF | 211 |
| 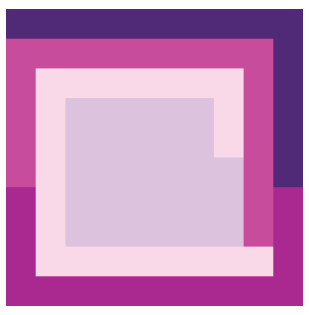 | Violet | #E4C2F4 | 228 |
| | | #B3009E | 179 |
| | | #4F107A | 79 |
| | | #FFD8F3 | 255 |
| | | #FF00E1 | 255 |
| 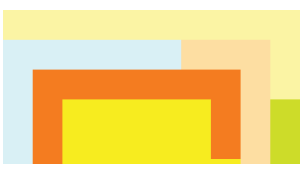 | Orange blossom | #FDDEA2 | 253 |
| | | #FF7D00 | 255 |
| | | #FFFFA1 | 255 |

| | | | |
|---|---|---|---|
| 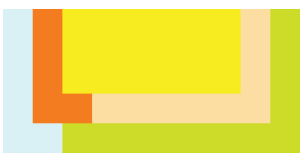 | Orange blossom | #FFFC02 | 255 |
| | | #9ECA3C | 158 |
| | | #D3FFFF | 211 |
| | | #FFFFFF | 255 |
| | | #FF928D | 255 |
| | | #FF7D00 | 255 |
| 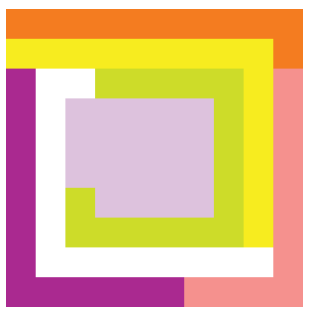 | Cut grass | #FFFC02 | 255 |
| | | #9ECA3C | 158 |
| | | #E4C2F4 | 228 |
| | | #FF00E1 | 255 |
| | | #FFFFA1 | 255 |
| | | #FFFC02 | 255 |
| 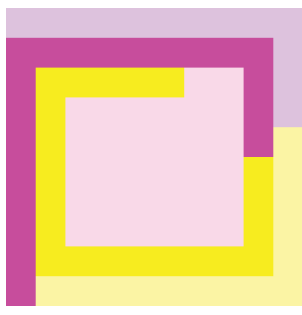 | Rose | #E4C2F4 | 228 |
| | | #FFD8F3 | 255 |
| | | #FF00E1 | 255 |

| G | B | relative area (%) |
|---|---|---|
| 255 | 202 | 22 |
| 183 | 72 | 34 |
| 255 | 255 | 20 |
| 176 | 240 | 15 |
| 202 | 60 | 9 |
| 202 | 60 | 14 |
| 194 | 244 | 48 |
| 16 | 122 | 21 |
| 0 | 225 | 17 |
| 255 | 161 | 16 |
| 252 | 2 | 12 |
| 223 | 31 | 18 |
| 202 | 60 | 24 |
| 183 | 72 | 17 |
| 255 | 255 | 13 |
| 194 | 244 | 28 |
| 0 | 158 | 16 |
| 16 | 122 | 15 |
| 216 | 243 | 22 |
| 0 | 225 | 19 |
| 222 | 162 | 15 |
| 125 | 0 | 16 |
| 255 | 161 | 12 |

| | | |
|---|---|---|
| 252 | 2 | 28 |
| 202 | 60 | 14 |
| 255 | 255 | 15 |
| 255 | 255 | 15 |
| 146 | 141 | 11 |
| 125 | 0 | 11 |
| 252 | 2 | 15 |
| 202 | 60 | 16 |
| 194 | 244 | 19 |
| 0 | 225 | 13 |
| 255 | 161 | 14 |
| 252 | 2 | 21 |
| 194 | 244 | 13 |
| 216 | 243 | 32 |
| 0 | 225 | 20 |
