# Supplementary material for: Shedding light on imagined scents: an fNIRS feasibility study of olfactory mental imagery and semantic cueing
Source: Front Neurosci. 2026 Aug 10;20:1825171. doi: 10.3389/fnins.2026.1825171 (PMC13500555; doi:10.3389/fnins.2026.1825171)
Supplement: Supplementary file 3 [file Data_Sheet_3.pdf]

Optode	X	Y	Z
D1	-48.325	67.493	20.901
D2	-54.498	68.788	-18.293
D3	-68.068	44.305	11.188
D4	-69.201	16.902	48.071
D5	-79.158	13.916	-12.285
D6	-83.651	-12.661	27.028
D7	-73.389	-40.987	61.743
D8	-44.547	-68.002	82.327
D9	48.522	67.016	21.782
D10	54.021	69.084	-17.587
D11	68.202	43.982	11.533
D12	69.152	18.343	46.92
D13	78.999	14.902	-11.581
D14	82.915	-10.604	29.84
D15	73.67	-40.111	61.571
D16	44.181	-67.297	82.983
S1	-35.441	80.614	-4.473
S2	-58.714	44.791	39.855
S3	-70.55	42.879	-19.263
S4	-78.428	15.42	18.947
S5	-74.677	-10.925	56.591
S6	-82.996	-43.839	31.464
S7	-52.679	-39.783	85.094
S8	-64.268	-70.38	58.921
S9	35.678	80.452	-4.2
S10	58.469	44.968	40.143
S11	70.103	43.768	-18.472
S12	78.115	16.208	20.212
S13	74.221	-10.2	57.017
S14	83.231	-40.985	33.593
S15	52.703	-38.84	85.18
S16	64.155	-68.949	60.453
